# Diversification of small RNA pathways underlies germline RNA interference incompetence in wild *Caenorhabditis elegans* strains

**DOI:** 10.1093/genetics/iyad191

**Published:** 2023-10-22

**Authors:** Han Ting Chou, Francisco Valencia, Jacqueline C Alexander, Avery Davis Bell, Diptodip Deb, Daniel A Pollard, Annalise B Paaby

**Affiliations:** School of Biological Sciences, Georgia Institute of Technology, Atlanta, GA 30332, USA; School of Biological Sciences, Georgia Institute of Technology, Atlanta, GA 30332, USA; School of Biological Sciences, Georgia Institute of Technology, Atlanta, GA 30332, USA; Department of Microbiology, University of Washington, Seattle, WA 98109, USA; School of Biological Sciences, Georgia Institute of Technology, Atlanta, GA 30332, USA; School of Biological Sciences, Georgia Institute of Technology, Atlanta, GA 30332, USA; Janelia Research Campus, Ashburn, VA 20147, USA; Department of Biology, Western Washington University, Bellingham, WA 98225, USA; School of Biological Sciences, Georgia Institute of Technology, Atlanta, GA 30332, USA

**Keywords:** *Caenorhabditis elegans*, RNA interference, natural genetic variation

## Abstract

The discovery that experimental delivery of dsRNA can induce gene silencing at target genes revolutionized genetics research, by both uncovering essential biological processes and creating new tools for developmental geneticists. However, the efficacy of exogenous RNA interference (RNAi) varies dramatically within the *Caenorhabditis elegans* natural population, raising questions about our understanding of RNAi in the lab relative to its activity and significance in nature. Here, we investigate why some wild strains fail to mount a robust RNAi response to germline targets. We observe diversity in mechanism: in some strains, the response is stochastic, either on or off among individuals, while in others, the response is consistent but delayed. Increased activity of the Argonaute PPW-1, which is required for germline RNAi in the laboratory strain N2, rescues the response in some strains but dampens it further in others. Among wild strains, genes known to mediate RNAi exhibited very high expression variation relative to other genes in the genome as well as allelic divergence and strain-specific instances of pseudogenization at the sequence level. Our results demonstrate functional diversification in the small RNA pathways in *C. elegans* and suggest that RNAi processes are evolving rapidly and dynamically in nature.

## Introduction

In *Caenorhabditis elegans*, the ability to silence genes by feeding worms *Escherichia coli* bacteria engineered to express RNA matching worm gene targets transformed molecular and developmental genetics (Timmons and Fire 1998; Kamath et al. 2003; Rual et al. 2004). However, even as *C. elegans* sits at the epicenter of research into gene silencing by small RNAs, wild strains vary significantly in capacity for RNA interference (RNAi). The universal laboratory strain N2 is robustly sensitive to RNAi, but its competence is not representative (Félix 2008). For example, RNAi against germline targets in several dozen wild strains revealed a range of responses, from negligible to more sensitive than N2 (Paaby et al. 2015). Wild strains also vary in competence for targets in the soma, and some strains show incompetence for RNAi by both feeding and injection (Tijsterman et al. 2002; Félix et al. 2011; Paaby et al. 2015). To date, the only causal variant identified for natural differences in RNAi is a frameshift lesion in the Argonaute *ppw-1*, which explains germline RNAi insensitivity in the Hawaiian strain CB4856 (Tijsterman et al. 2002). The genetics underlying differences in RNAi efficacy in wild *C. elegans* are otherwise unknown.

The umbrella term “RNA interference” describes the general mechanism of gene silencing via dsRNA (Yigit et al. 2006) and includes the microRNA (miRNA), short interfering RNA (siRNA), and PIWI-interacting (piRNA) pathways. These pathways overlap in gene set and molecular mechanisms but mediate processes as diverse as cell growth and tissue differentiation, adaptive immunity against pathogens, transgenerational epigenetic inheritance, and germline defense against transposons (Grishok 2013; Wilson and Doudna 2013). Thus, gene knockdown in the lab via exogenous delivery of sequence-specific dsRNA is possible because of the native, complex meta-phenomenon of gene regulation by small RNAs that dominates *C. elegans* biology (Youngman and Claycomb 2014; Houri-Zeevi et al. 2020).

All RNAi processes induce gene silencing via the association of small RNAs with Argonaute effector proteins (Wilson and Doudna 2013); the Argonaute superfamily includes the ancient AGO proteins, the PIWI Argonautes in animals, and in *C. elegans*, the nematode-specific WAGO proteins (Youngman and Claycomb 2014). Many of the genes that encode RNAi machinery are shared across plants, animals, and fungi and appear deeply conserved within the eukaryotic lineage (Shabalina and Koonin 2008; Wynant et al. 2017). Yet, RNAi processes also appear fast-evolving within and across eukaryotic taxa. The ability to silence genes by dsRNA appears intermittently and shows evidence of rapid evolution within the *Caenorhabditis* genus (Winston et al. 2007; Nuez and Félix 2012), across nematodes generally (Dalzell et al. 2011; Buck and Blaxter 2013), and in other systems (Obbard et al. 2009). Argonautes and associated RNAi factors also exhibit taxon-specific patterns of gene duplication, loss, and diversification, likely representative of diversification of biological functions (Obbard et al. 2009; Dalzell et al. 2011; Buck and Blaxter 2013); the WAGO expansion in nematodes has been hypothesized to underlie the extraordinary diversification of worms across environments, perhaps having enabled adaptations associated with environmental sensing, parasitism, and immunity (Buck and Blaxter 2013). Thus, intraspecific variation in RNAi competence in *C. elegans* mirrors lability in RNAi observed over long timescales (Nuez and Félix 2012) and raises questions about contemporaneous selection pressures.

Given the centrality of RNAi in *C. elegans*, why is its efficacy so variable? To begin to explore this question, here, we investigate the genetic basis of natural variation in RNAi by focusing on wild strains that are deficient in germline RNAi. First, we improve readout of the RNAi response by measuring effects at the molecular level and over organismal lifespan. We then ask whether RNAi incompetence involves genes beyond *ppw-1*, whether the genetic architecture of incompetence is simple or complex, and whether causal variants are shared or unique among strains. At the population level, we evaluate expression variation and allelic diversity at genes known to mediate RNAi, to compare RNAi responses at the organismal level to proximate causes of failure. These analyses uncover evidence of extensive diversification of RNAi activity within *C. elegans*, consistent with rapid and recent evolution of a genetically complex trait. This level of functional variability in RNAi pathways offers a useful access point into connecting the vast body of *C. elegans* RNAi research to the biological relevance of these processes in nature.

## Materials and methods

### Strains used in this study

Supplementary Table 7 contains a complete list of strains used in this study. To select wild strains putatively incompetent for germline RNAi, we first considered those exhibiting poor responses in prior studies (Tjisterman et al. 2002; Paaby et al. 2015). We also screened untested, highly diverged strains (Cook et al. 2017) on *par-1* and *pos-1* RNAi to sample the population more broadly. Those preliminary results are not included in this study, as our aim was to define a set of candidate strains to then quantify with carefully controlled methodology. The final set of 7 strains was chosen based on weakest responses. We verified that these strains were substantially diverged from each other and broadly represented nucleotide diversity across the species by identifying their placement on the species tree (Supplementary Fig. 9) (Cook et al. 2017). To introduce germline-expressed GFP into wild strains, we introgressed *zuIs178 [his-72(1 kb 5′ UTR)::his-72::SRPVAT::GFP::his-72 (1KB 3′ UTR) + 5.7 kb XbaI − HindIII unc-119(+)]; stIs10024 [pie-1::H2B::GFP::pie-1 3′ UTR + unc-119(+)]* into strains CB4856, ECA369, JU1522, and QX1211 by crossing to RW10029 and backcrossing to the wild strain for 10–18 generations.

### Worm husbandry

Worms were cultured following standard protocol (Stiernagle 2006), though we added 1.25% agarose to plates used to maintain non-N2 wild strains, to avoid burrowing. Worms were maintained at 20°C without starving for at least 3 generations before initiating an experiment, with the exception of QX1211, which was maintained at 18°C to avoid induction of the mortal germline phenotype (Frézal et al. 2018).

### RNA interference

#### General culture conditions

RNAi was induced by feeding and experiments were carried out on plates, at 20°C, based on methods previously described (Kamath et al. 2001; Ahringer 2006). In brief: to target endogenous germline-expressed genes, we fed worms HT115  *E. coli* bacteria that had been transformed with the pL4440-derived *par-1* (H39E23.1), *par-4* (Y59A8B.14), *pos-1* (F52E1.1), or GFP feeding vector (Timmons et al. 2001). The *par-1* and *pos-1* vectors were obtained from the Ahringer feeding library (Kamath and Ahringer 2003); *par-4* was a gift from Miyeko Mana. To target GFP, we transformed HT115 with pL4417, which carries 0.7 kb of GFP coding sequence (Timmons et al. 2001). We used *E. coli* carrying the empty pL4440 vector as a negative control. Bacteria were streaked from frozen stocks onto LB agar plates with carbenicillin (25 ug/mL) and tetracycline (12.5 mg/mL); liquid cultures were inoculated with 5–10 colonies from <1-week-old plates, into LB broth with carbenicillin (50 ug/mL) and tetracycline (12.5 mg/mL), incubated for 16–18 h shaking at 37°C, and then amplified in a 1:200 dilution with carbenicillin (50 ug/mL) for 6 h. Seeded plates were incubated in the dark at room temperature and used no earlier than 44 h and no later than 78 h. Experimental worms were exposed to RNAi bacteria as L1s by hatching on RNAi plates, synchronized either by bleaching (Stiernagle 2006) or by timed egg-laying by the hermaphrodite mothers.

We note that in our hands, RNAi phenotypes of wild strains appear exquisitely sensitive to experimental conditions. We recommend rigorous control of temperature, humidity (including plate age), and bacterial culture, as well as parental age across strains, which may otherwise vary in developmental timing, and to consider potential transgenerational effects that may confound RNAi phenotypes over time or between strains, such as the mortal germline (Frézal et al. 2018).

#### Embryonic lethality assays

**Fig. 1.**
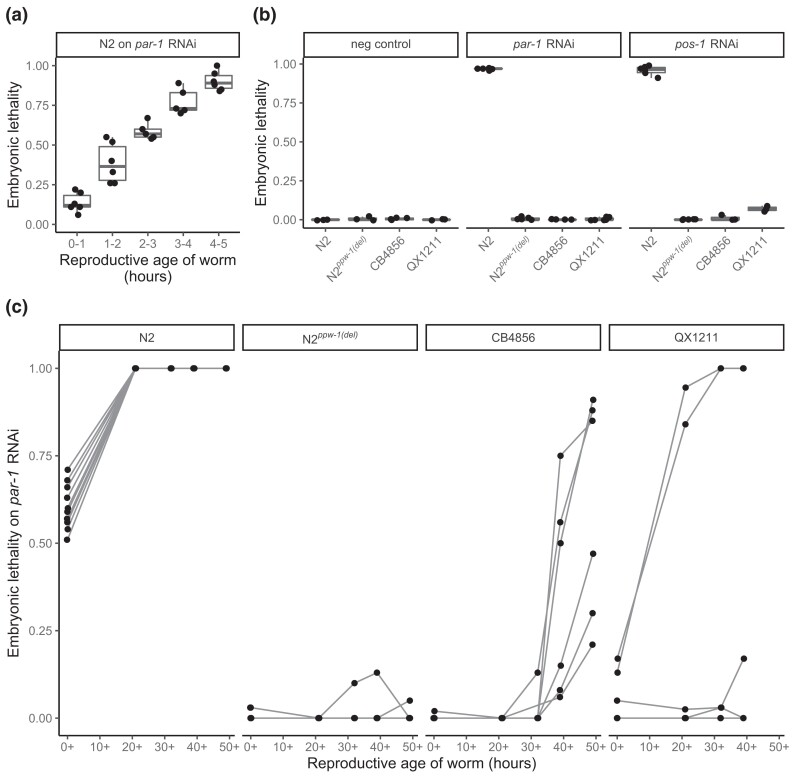
Embryonic lethality following RNAi against germline-expressed targets. a) Lethality following *par-1* RNAi is incompletely penetrant in the earliest-laid N2 embryos, even though parental worms were exposed to the feeding vector since hatching. b) Offspring from worms several hours into reproductive maturity show nearly complete embryonic lethality in N2 following *par-1* and *pos-1* RNAi but negligible penetrance in the N2*^ppw-1(del)^* mutant and in wild strains CB4856 and QX1211. c) To assess the germline RNAi response more comprehensively, embryonic lethality was scored for individuals over the entire reproductive lifespan. Each point represents the proportion of dead embryos, out of total laid on a plate by a single hermaphrodite, in the given time interval. The data include all offspring of all hermaphrodite mothers; time intervals were chosen to space out the number of offspring per plate (∼30–100); the *x*-axis labels indicate the approximate midpoint of the time intervals. Connecting lines indicate each worm's progeny over time. Embryonic lethality for all strains on the negative control empty vector was negligible (data not shown). Brood sizes were similar across strains (Supplementary Fig. 1).

**Fig. 2.**
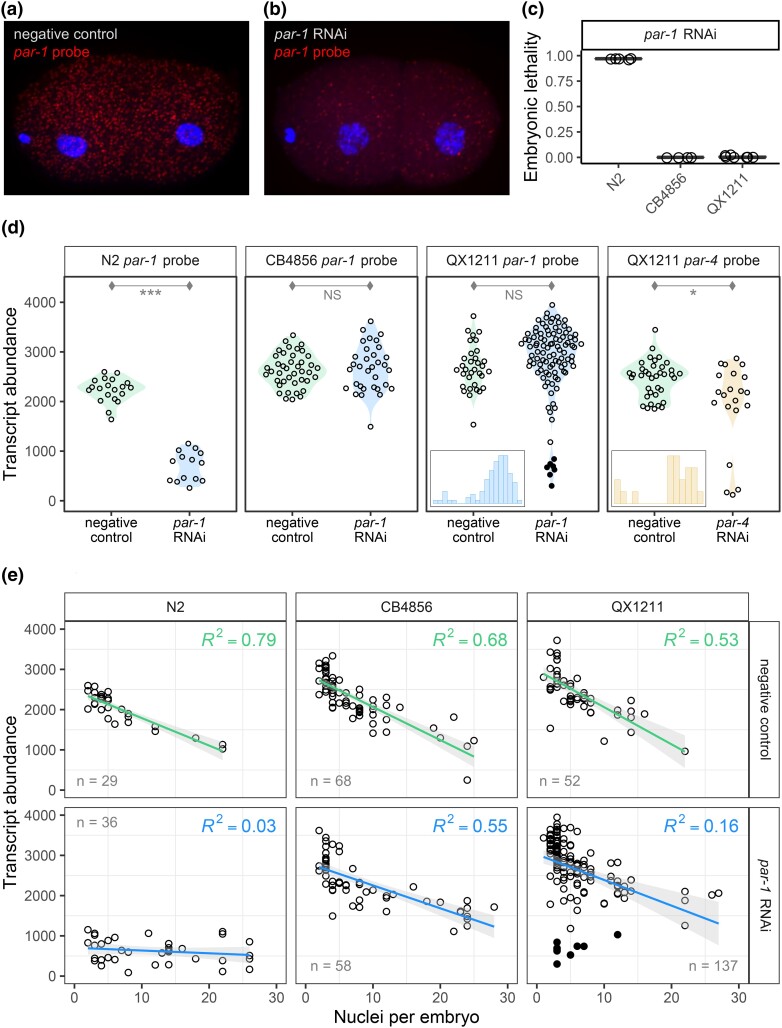
Transcript abundance in individual RNAi-treated and untreated embryos, visualized via single-molecule fluorescence in situ hybridization (smFISH). a and b) Representative embryos are shown from strain N2, from a mother reared in the control condition or with RNAi against *par-1*; spots indicate *par-1* transcripts, and DAPI staining shows nuclei, which were used to identify embryo stage. c) Embryonic lethality was simultaneously measured in matched samples. To limit variation due to reproductive age of the mothers, we collected embryos from a tightly controlled time window in early reproduction. d) Transcript abundance for early-stage embryos (up to 4 cells). Insets show transcript count histograms for the treated *par-1* and *par-4*  QX1211 embryos, which suggest bimodality by an excess mass test (*P* < 0.01 for both). e) Transcript abundance for *par-1* for the same experiment but now including later stage embryos with up to 30 nuclei. Green (negative control) and blue (RNAi treatment) lines indicate the linear regression of transcript counts onto embryonic stage; gray shading indicates the 90% confidence interval. For d and e), each point represents one embryo, and the dark QX1211 points highlight the *par-1*-treated embryos with counts at or below those of N2. Significance levels (*t*-tests): *P* < 0.001 (***), *P* < 0.01 (**), and *P* < 0.05 (*).

**Fig. 3.**
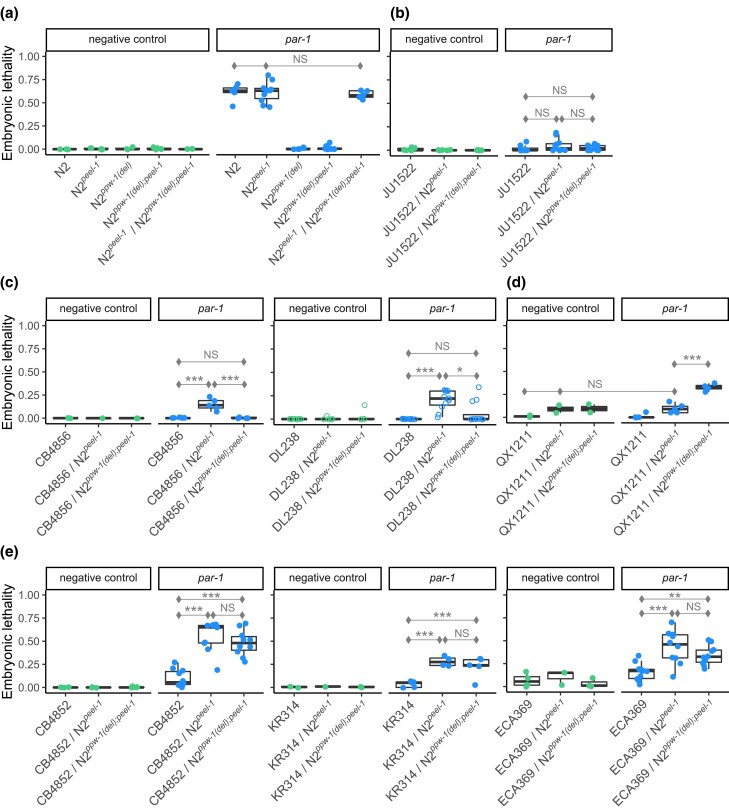
Complementation tests between 7 wild strains with low RNAi response and the RNAi-sensitive laboratory strain N2, with and without the *ppw-1* deletion allele. Response was measured by embryonic lethality following RNAi by feeding against the embryonic target *par-1*. Points represent the average across pooled hermaphrodites, with one exception (see below). a) To circumvent embryonic lethality arising from the *zeel-1;peel-1* genetic incompatibility (Seidel et al. 2008, 2011), we used a null allele of the sperm-delivered toxin *peel-1* in N2, which has no effect on RNAi in either the responsive (N2) or the resistant (N2*^ppw-1(del)^*) backgrounds. A single copy of *ppw-1* is sufficient to fully restore the germline RNAi response in N2. b–e) Complementation tests for 7 wild strains with weak germline RNAi, representing a diversity of genetic backgrounds. For DL238 (c), the open circles represent the proportion of dead embryos per individual; the overall pattern qualitatively replicates that which we observed in pooled hermaphrodites (Supplementary Fig. 2; individuals shown here to highlight variability). For QX1211 (d), the *sup-35;pha-1* incompatibility (Ben-David et al. 2017) induced embryonic lethality, visible in the control condition and the genotype without the *ppw-1* deletion in the *par-1* treatment (see Supplementary File 1 for details). Significance levels (Tukey's contrasts): *P* < 0.001 (***), *P* < 0.01 (**), and *P* < 0.05 (*).

**Fig. 4.**
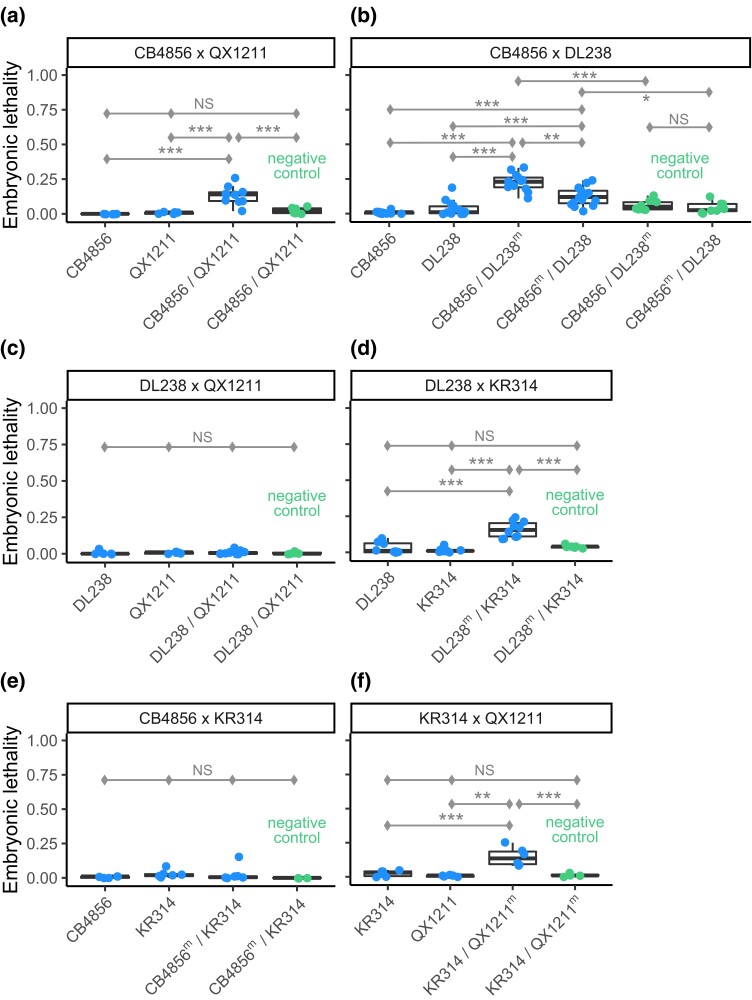
Pairwise complementation tests between 4 wild strains with low RNAi response. a–f) Response was measured by embryonic lethality following RNAi by feeding against the embryonic target *par-1*. The heterozygote genotypes were generated by crossing males and hermaphrodites in both directions (a–c), except for crosses with strain KR314, which does not produce fertile males (d–f). With the exception of CB4856 × DL238 (b), in which hermaphrodites sired by DL238 exhibited a significantly stronger response than those sired by CB4856, cross direction had no effect on embryonic lethality in the next generation and plots show pooled data. Significance levels (Tukey's contrasts): *P* < 0.001 (***), *P* < 0.01 (**), and *P* < 0.05 (*).

To measure RNAi response by phenotypic penetrance, we targeted *par-1* or *pos-1* transcripts in the hermaphrodite germline and measured embryonic lethality in the next generation. Experimental worms were reared on RNAi plates and transferred as L4s to fresh RNAi plates for the egg-laying assay, remaining continuously exposed to RNAi bacteria since their hatching. For all experiments except those explicitly testing variation in penetrance between individual worms (Figs. 1b and Fig. 3c), the L4 hermaphrodites were pooled in small groups of 4–6 on 6–10 replicate assay plates. For the complementation tests (Figs. 3 and 4, Supplementary Fig. 2), mated hermaphrodites of the parental generation were permitted to lay on RNAi plates, and the F1 genotypes of the selected hermaphrodites were verified by the presence of ∼50% male offspring; in the assay, all embryos within the first ∼15 h of egg-laying were scored for hatching, typically 100–200 embryos per plate. For assays testing RNAi within a defined window of reproductive maturity, we scored the embryos laid in a 4–6-h window within the first 8 h of egg-laying (Fig. 1a) or a 2-h window 4 h after egg-laying began (Fig. 2c). For the experiments measuring RNAi in individual worms over their reproductive lifespan (Fig. 1b, Supplementary Figs. 5 and 10), the L4 hermaphrodites were singled to RNAi plates, permitted to lay embryos, and continually transferred to fresh plates until they ceased to lay or laid only unfertilized eggs. To score embryos as dead or alive, we removed the egg-laying adult(s), incubated the plates at 20°C for 24 h, counted (dead) embryos, and hatched larvae using a stereoscope. Experiments included 6–10 (RNAi treatment) or 4–6 (negative control) replicate plates.

In the experiment following individual worms over their lifespan (Fig. 1b), we tested for differences in RNAi among low-response strains by modeling embryonic lethality in CB4856, QX1211, and N2*^ppw-1(del)^* with a linear model of the form


$$\begin{array}{rl} E(Y)\;= & \beta 0+\beta 1X\mathrm{strain}+\beta 2X\mathrm{age}+\beta 3X\mathrm{strain}*X\mathrm{age}\\ & +\beta 4X\mathrm{worm}(\mathrm{strain})+\varepsilon , \end{array}$$

which includes the main effects of strain and worm age, the interaction between them, and the effect of individual worms nested within strain. To test the specific hypothesis that individual worms varied within CB4856 and QX1211, we employed a reduced model considering only age and individual worm on strain-specific data. For QX1211, post-hoc tests between individual worms were performed with the function *glht* in the R package *multcomp* (Hothorn et al. 2008). To test the effect of genotype on embryonic lethality following exposure to RNAi in the complementation tests (Figs. 3 and 4), the counts of dead embryos and hatched larvae from each replicate plate were bound together as a single response variable and modeled with a generalized linear model with a quasibinomial error structure, implemented by the *glm* function in R. The model included a single linear predictor for genotype and took the form $E(Y)=g^{-1}(\beta_{0}+\beta_{1}X_{\mathrm{genotype}})$ . Within each experiment, differences between specific genotypes were assessed by pairwise contrasts using the “Tukey” specification in the function *glht* in the R package *multcomp* (Hothorn et al. 2008).

#### RNAi against GFP

To measure germline RNAi by GFP knockdown, worms carrying a histone-linked GFP driven by a *pie-1* promoter were fed RNAi bacteria targeting GFP. Synchronized animals were grown on RNAi plates and then individually selected for imaging at the following stages: young adults (6 ± 2 h after exiting L4 stage), day 1 adults (24 ± 2 h), and day 2 adults (48 ± 2 h). For whole worm fluorescence imaging, animals were anesthetized with 10 mM NaN_3_ and mounted on 2% agarose pads and then imaged using a ×10 objective with the PerkinElmer UltraVIEW VoX spinning disk confocal microscope equipped with an EM-CCD camera. Raw images were exported as OME.TIFF files. We used Fiji (Schindelin et al. 2012) to acquire the sums of intensity in the Z projection and then quantitated the GFP fluorescence by subtracting the integrated intensity of the background, over the area of the worm, from the integrated intensity of the whole animal. To test whether RNAi-treated worms exhibited reduced fluorescence relative to control worms, we analyzed the 6 samples (3 treatment timepoints and 3 control timepoints) for each strain using a one-way ANOVA and then performed treatment–control contrasts within each timepoint using the R function *TukeyHSD()*.

### Single-molecule fluorescence in situ hybridization

#### Sample preparation and imaging

Custom Stellaris FISH probes were designed with the Stellaris Probe Designer (LGC Biosearch Technologies). We excluded polymorphic sites during probe design. Worms were synchronized on tryptone-free NGM agar plates at the L1 stage and reared on RNAi bacteria as described above. Embryos were extracted by standard bleaching/washing, fixed using 3.7% formaldehyde in RNase-free phosphate buffered saline, and hybridized (100 nM at 37°C for 4 h) with a Quasar 570 labeled probe set targeting either *par-1* or *par-4*, following the manufacturer's instructions. Samples were mounted using VECTASHIELD antifade mounting medium with DAPI (Vector Labs #H-1200) on no. 1 cover slides. Images were captured with a ×100 oil immersion objective on a PerkinElmer UltraVIEW VoX spinning disk confocal microscope equipped with an EM-CCD camera and piezoelectric motorized stage. Three-dimensional image stacks were collected using Volocity 3D visualization software (PerkinElmer) and exported as TIFF files.

#### Quantitative analysis

Image segmentation masks were applied, and chromosome clusters were counted using ImageJ (Schneider et al. 2012). Quantification of single-molecule FISH spots was performed using Aro, a MATLAB-based machine learning pipeline designed for single-molecule visualization in worm embryos (Wu and Rifkin 2015). The training sets for the random forest classifier were generated from multiple samples of each genetic background and treatment. To test whether means or variances in transcript counts differed for RNAi-treated vs untreated samples within a strain, we applied 2-sample *t*-tests and *F*-tests, respectively, to the N2 and CB4856 data. For QX1211, which showed clusters of low counts in both the *par-1* and *par-4* data, we tested those distributions for multimodality using the function *modetest* in the R package *multimode* (Ameijeiras-Alonso et al. 2021). To compare the treated and untreated QX1211 samples, we used a Mann–Whitney U nonparametric 2-sample test. For these tests in all 3 strains, we only considered early-stage embryos (up to 4 cells). To evaluate changes in transcript abundance over a wider range of embryonic development, we considered embryos with up to 30 nuclei and used ANCOVA to ask whether, adjusted for embryo stage, transcript levels varied across strains within the negative control condition and whether, adjusted for embryo stage, transcript levels varied between control and treatment conditions within a strain. We used minimal model selection to test for changes in the way transcript level depended upon embryo stage (i.e. changes in slope). We estimated ANCOVA effect sizes as ω^2^ using the R package *sjstats* (Lüdecke 2018).

### RNA-seq

#### Library preparation and sequencing

Healthy cultures of strains N2, CB4856, QX1211, JU1088, and EG4348, reared for several generations without starving or bleaching, were bleached to retrieve large numbers of embryos. Synchronized L1 larvae were reared on plates with the empty RNAi feeding vector, details as described above. Young, reproductively mature hermaphrodites were washed off plates and rinsed twice with M9, and then, RNA was extracted with TRIzol (Invitrogen #15596026) and RNeasy columns (Qiagen #74104), following (He 2011). All samples were collected and processed simultaneously and in triplicate, starting with replicate plates of worms. Libraries were prepared with the NEBNext Ultra II Directional RNA Library Prep Kit for Illumina (NEB #7760), with cDNA generated from fresh RNA samples using 500 ng of RNA and 10 cycles of PCR. Libraries were quality checked using an Agilent 2100 Bioanalyzer, and fragments were size-selected via BluePippon (Sage Science). Libraries were sequenced on an Illumina NextSeq for single-end 75 bp reads at the Molecular Evolution Core facility at the Georgia Institute of Technology.

#### Alignment and gene expression quantification

We generated strain-specific transcriptomes for RNA-seq read quantification by patching SNPs and indels from CeNDR (release 20210121) (Cook et al. 2017) onto the N2 reference genome (release ws276) (Harris et al. 2020) using *g2gtools* (v0.1.31 via conda v4.7.12, Python v2.7.16) (https://github.com/churchill-lab/g2gtools), followed by transcriptome extraction. Specifically, for each nonreference strain, indels were first chained onto the reference genome using *g2gtools vcf2chain* and SNPs were patched onto the reference genome FASTA using *g2gtools patch*. Next, indels were chained onto the SNP-patched genome using *g2gtools transform* and strain-specific GTFs were created from this updated genome FASTA via *g2gtools convert*. Strain-specific transcriptomes were generated from these strain-specific genome FASTAs and GTFs using *gffread* (v0.12.7) (Pertea and Pertea 2020).

Transcript-level quantification was performed using Salmon (v1.4.0) (Patro et al. 2017). Before Salmon quantification, Illumina TruSeq adapters were trimmed from RNA-seq reads using Trimmomatic (v0.3.9) (Bolger et al. 2014) with parameters *ILLUMINACLIP:TruSeq3-SE.fa:1:30:12*. Salmon index files were built from the strain-specific transcriptomes using command *salmon index* with options *-k 31 --keepDuplicates* (all others default; no decoy was used). Transcript quantification was performed with *salmon quant* with options *-l SR --dumpEq*, *--rangeFactorizationBins 4*, *--seqBias*, and *--gcBias* and the library-specific fragment length arguments *--fldMean* and *--fldSD*.

#### Analysis of gene expression

We performed all expression analyses in R (v4.0.3) (R Core Team 2021) using data processed with the DESeq2 package (v1.32.0) (Love et al. 2014). We used the *tximport* package (v1.20.0) (Soneson et al. 2015) to import Salmon transcript quantification data into DESeq2 and to compute gene-level expression quantification estimates. Genes with 10 or fewer counts total across all samples after *tximport* were excluded from downstream analysis (18,589 genes retained).

To test for differential expression across strains, gene counts were modeled using the negative binomial generalized linear model in DESeq2:


$$\log2(q_{ij})=\beta_{i}x_{j},$$

where for gene *i* and sample *j*, *q* is proportional to the true concentration of RNA fragments for the gene. β*_i_* gives the log2 fold changes for gene *i*, and *x* represents the strain; batch was not included in the model because all samples (3 biological replicates per strain, 5 strains) were processed simultaneously. Significance testing for differential expression was performed by likelihood ratio test (LRT) in DESeq2, which captured strain-wise significance by comparing the above model to a reduced model containing only the intercept (Love et al. 2014). Genes were considered differentially expressed by strain if the genome-wide adjusted *P*-value (FDR) from the LRT was <0.1 (5,464 of the 18,589 genes passed this threshold overall). Estimates of differential expression between N2 and each other strain were extracted via pairwise contrasts; effect sizes and *P*-values were corrected using the “*ashr*” method from the *ashr* package (v2.2-47) (Stephens 2017).

We assessed strain-wise variance per gene by first obtaining normalized gene expression data from the variance-stabilizing transformation (*vst* function) in DESeq2. This transformation puts the data in log2 scale, incorporates library size and gene length normalizations, and makes the variance independent of the mean (Love et al. 2014). Strain-wise variance for each gene was then estimated by one-way ANOVA, i.e. *counts ∼ strain*; the sums of squares for the strain term were extracted using the *aov* function.

Expression-matched gene sets for the RNAi genes were constructed by first identifying, for each RNAi gene, all genes with *vst*-normalized mean expression (across all samples) within one percentile of the RNAi gene's mean expression (+/−0.005 in expression quartile). One of these genes was chosen at random for each RNAi gene, and this procedure was repeated 10,000 times to obtain the 10,000 random expression-matched gene sets. The median strain-wise variance (of *vst*-normalized gene counts) for each random set of genes was computed and compared with the median strain-wise variance of the RNAi genes.

### Droplet digital PCR

To design an unbiased primer set, we identified regions of identical sequence between *ppw-1* and *sago-2* and across the 10 strains of interest. Following Kamitaki et al. (2018), we chose primers to target both genes and probes to discriminate between *ppw-1* (FAM) and *sago-2* (HEX). Sequences are as follows: forward (CTTGGTACCGCTCCGCTC), reverse (GCTGATTCGGTTTGATCGTC), *ppw-1* probe (AGACGAGAAATGTGGAGAGGGGAA), *sago-2* probe (AGACGAGAAATGAGGAGTGGGGAA). Both probes anneal in the same location, ensuring competition between them.

Worms from strains N2, CB4856, CB4852, DL238, ECA369, EG4348, JU1088, JU1581, KR314, and QX1211 were reared under standard conditions (as above), bleached to isolate embryos, and grown to reproductive maturity. RNA was extracted with TRIzol (Invitrogen #15596026) and RNeasy columns (Qiagen #74104), following (He 2011). RNA was collected at 2 timepoints, early and middle reproductive maturity (68 ± 2 h and 90 ± 2 h after bleaching, respectively). RNA sample concentrations were quantified and standardized using a NanoDrop (Thermo Scientific), and cDNA was synthesized using the ProtoScript II First Stand cDNA Synthesis Kit (NEB #E6560S). The experiment was replicated as follows: from each experimental condition, we collected 2 RNA samples, for 2 biological replicates; within the plate, each reaction was duplicated, for 2 technical replicates; and we conducted the entire experiment twice.

Droplet digital PCR was carried out with the Bio-Rad QX200 system following the manufacturer's protocol, and results were obtained using the QuantaSoft software (Bio-Rad), via automatic thresholding followed by manual confirmation of droplet selection. All samples produced >8,000 droplets and results from all samples were retained. Concentration, given by number of copies per μL, was modeled with a quasipoisson error structure using the *glm()* function in R. As *ppw-1* was detected at an order of magnitude higher than *sago-2*, we analyzed the 2 genes separately. By model selection, we identified the minimal model that best described the observed differences in concentration For the *ppw-1* analysis, we dropped run date from the model, as it was not significant; for *sago-2*, run date contributed <1% to the total observed deviance (Supplementary Table 3) but was nevertheless significant, so it was retained. The final models were Concentration ∼ Strain*DevStage/BiolRep for *ppw-1* and Concentration ∼ RunDate + Strain*DevStage/BiolRep for *sago-2*. To determine which strains differed in *ppw-1* or *sago-2* levels, we performed pairwise contrasts among strains using the *TukeyHSD()* function and a family-wise confidence level of 95% (only a subset of comparisons are reported in the text). To determine which strains showed differences in concentration according to developmental stage, we performed pairwise contrasts using the *lsmeans()* function in the package *lsmeans*, using a confidence level of 95% following a Bonferroni correction for multiple tests.

### Genotype and sequence analysis

To evaluate population-level allelic variation at known RNAi genes, we queried the *C. elegans* Natural Diversity Resource (https://elegansvariation.org), which provides genotype data for 403 wild isotypes from short-read sequence data mapped to the N2 reference genome (Cook et al. 2017). Specifically, we downloaded the VCF (WI.20200815.hard-filter.isotype.vcf) and used the R package *VariantAnnotation* (v1.38.0) (Obenchain et al. 2014) to extract information about mapping coverage and mutations, including SnpEff-defined impact classifications, and the package *PopGenome* (v2.7.5) (Pfeifer et al. 2014) to estimate nucleotide diversity, at each gene. Differences in nucleotide diversity and variant counts between RNAi genes and all other protein-coding genes in the genome were assessed by 2-tailed Mann–Whitney U tests. For individual genes, haplotype networks and haplotype diversities (Nei and Tajima 1981) were determined using the R package *pegas* (v1.0-1) (Paradis 2010).

For a subset of strains, we verified and/or supplemented the genotype data with de novo-assembled genome data and long-read data. Genomic DNA of strains AB2, EG4347, EG4348, JU1088, JU1171, PB306, PX174, QX1211, and QX1216 was prepared using standard phenol/chloroform extraction and ethanol precipitation. Samples were cleaned with DNA Clean & Concentrator columns (Zymo Research #D4004), and libraries were prepared using NEBNext Ultra II FS DNA Library Prep Kit for Illumina (New England Biolabs #E7805) and Multiplex Oligos for Illumina (NEB #E7500), with customized fragmentation and purification steps to enrich for desired sizes. A final DNA size selection targeting 650bp ± 50 bp was performed using BluePippin (Sage Science). The libraries were sequenced on a HiSeq 2500 (Illumina) on Rapid Run Mode (paired-end 2 × 250 bp) in the Molecular Evolution Core at the Georgia Institute of Technology. Raw data were trimmed using Cutadapt (v1.18) (Martin 2011), and quality control was performed with FastQC (Andrews 2021). Reads were then assembled into contigs with DISCOVAR de novo (v52488) (Broad Institute) using default parameters. Separately, QX1211 and JU1088 genomic DNA samples were snap frozen with liquid nitrogen and sent to the Georgia Genomics and Bioinformatics Core (GGBC) at the University of Georgia. Quality was assessed by Qubit (Invitrogen) and NanoDrop (Thermo Scientific), molecular weight distribution was assessed by fragment analysis, and sizes >15 kb were selected by BluePippin (Sage Science). Each sample was sequenced on a single SMRT Cell on the PacBio Sequel I platform (Pacific Biosciences). Genome assembly was performed by the GGBC using Canu (v1.7) (Koren et al. 2017).

### Computing

Unless otherwise specified, all analyses were performed in R (R Core Team 2021), and figures were generated with the packages *ggplot2* (Wickham 2016) and *ggpubr* (Kassambara 2020). Computationally intensive jobs, including read mapping and genome assembly, were performed on the Partnership for an Advanced Computing Environment (PACE), the high-performance computing platform at the Georgia Institute of Technology.

## Results

### Germline RNAi varies in expressivity and penetrance over reproductive age and among genotypes

Prior work examining embryonic gene knockdown in wild *C. elegans* demonstrated that strains vary quantitatively in the strength of their germline RNAi response and that strains CB4856 and QX1211 appear largely incompetent for germline RNAi (Paaby et al. 2015). In contrast, the common wild-type laboratory strain N2 is highly sensitive to RNAi, though germline RNAi can be eliminated in N2 with a deletion at the WAGO Argonaute *ppw-1* (Tijsterman et al. 2002; Yigit et al. 2006). Incompetence in CB4856 is mostly explained by loss of function at *ppw-1*, specifically a naturally occurring mutation encoding a frameshift and early stop upstream of the critical PAZ and PIWI domains, though analyses have also indicated genetic complexity beyond *ppw-1* in mediating germline RNAi in this strain (Tijsterman et al. 2002; Elvin et al. 2011; Pollard and Rockman 2013). To date, this mutation in CB4856 is the only known causal variant for natural variation in *C. elegans* RNAi response.

In this study, we sought to investigate the genetic basis of germline RNAi deficiency in wild strains. First, to establish a baseline of comparison, we used a standard assay to phenotype the RNAi response in CB4856, QX1211, N2, and N2 mutants (N2*^ppw-1(del)^*). We fed worms *E. coli* expressing dsRNA targeting the maternal-effect, embryonic-required genes *par-1* and *pos-1*, which have commonly been used to measure germline RNAi (Tijsterman et al. 2002; Elvin et al. 2011; Pollard and Rockman 2013), and then counted dead embryos in the next generation. Since the penetrance of RNAi phenotypes depends on worm age (Fig. 1a), conventional approaches typically score embryos from worms well into reproductive maturity (Kamath et al. 2001; Pollard and Rockman 2013). We likewise assayed embryos from worms starting several hours into reproductive maturation, and our observations were consistent with prior reports: wild-type N2 exhibited high lethality, and the 3 incompetent strains exhibited very low or negligible lethality (Fig. 1b).

However, we reasoned that restricting the RNAi phenotype to a specific age window might obscure differences among strains. To next evaluate how the response changes over time in the strains, we scored the penetrance of embryonic lethality over the complete reproductive lifespan. We conducted this assay on individuals, providing a time series of responses for each egg-laying animal. In this assay, we targeted *par-1*, which provides the more sensitive readout since it is not as lethal. Here, each of the 3 incompetent strains exhibited a distinct response, indicating differences in genetic mechanism (Fig. 1c).

N2 showed complete lethality in all but the earliest offspring, suggesting that in this sensitive strain, early amplification of the initial trigger rapidly induces total gene knockdown. In the mutant N2*^ppw-1(del)^*, however, nearly all embryos hatched, including late-age embryos (Fig. 1c), indicating that the loss of *ppw-1* is not compensated by other genes in the N2 background.

In CB4856, hermaphrodite mothers exhibited no evidence of an RNAi response in the first half of their reproductive lifespan, but embryonic lethality emerged in the second half and increased with parental age (Fig. 1c). This suggests that the mutation in *ppw-1* is either not a null allele or permits some PPW-1 activity or that other genes in the CB4856 background partially compensate for the loss of PPW-1, promoting a delayed RNAi response.

QX1211 exhibited a third unique noncompetent response. After a short delay, embryonic lethality was either negligible or complete, suggesting that RNAi in QX1211 is either “on” or “off” in individual animals (Fig. 1c). Thus, unlike N2*^ppw-1(del)^*, in which the RNAi response appears abolished, CB4856 and QX1211 do exhibit limited responses but with distinct patterns of activity: in CB4856, the response is delayed and incomplete; in QX1211, it is partially delayed, with higher expressivity and variable penetrance.

The distinctions among the 3 low-response strains are supported by statistical analysis. By linear model, we considered strain, reproductive age, the interaction between strain and age, and individual worm; all effects were significant (*P* < 0.001 for all; Supplementary Table 1), indicating that RNAi varies among strains and over reproductive age and that the timing of the response is specific to strain. Moreover, individual QX1211 worms exhibited distinct responses but CB4856 worms did not, supporting the conclusion that QX1211 is capable of alternate phenotypes. Specifically, each QX1211 “on” worm was significantly different from each “off” worm but not other “on” worms (Fig. 1b) and vice versa (*P* < 0.01 for all, Tukey's contrasts). These results point to distinct differences in the execution of germline RNAi within *C. elegans*. However, the use of an end-point phenotype to read out the RNAi response, i.e. embryonic lethality, does not capture activity at the molecular or cellular level. Moreover, variation in the *par-1* pathway between strains might influence phenotypic expression, confounding interpretation of the RNAi response (Paaby et al. 2015). Therefore, we developed an assay to measure the expression and knockdown of the target gene directly.

### Target transcript knockdown confirms distinct RNAi responses across strains

To assess how the RNAi response varies among strains at the molecular level, we used single-molecule fluorescence in situ hybridization (smFISH) to visualize transcripts of germline RNAi targets at high spatial and temporal resolution within embryos. Since smFISH visualizes individual molecules via hybridization of dozens of oligonucleotide probes, which in aggregate produce a detectable fluorescent spot (Raj et al. 2008), it captures signals from intact RNAs, not those degraded by RNAi.

We examined *par-1* transcript levels in *par-1* RNAi-treated and RNAi-untreated embryos of N2, CB4856, and QX1211. We collected embryos from gravid worms in early reproductive maturity, in a narrow 2-h window, to maximize precision in estimating the RNAi response. At this timepoint, many *par-1* transcripts are degraded in RNAi-treated N2 embryos (Fig. 2a and b).

Treated N2 embryos of this timepoint go on to show complete lethality, but in CB4856 and QX1211, lethality is negligible (Fig. 2c).

All 3 strains displayed robust expression of the target gene in untreated embryos (Fig. 2d), indicating that levels of native gene expression are unlikely to be a major influence on lethality penetrance. However, in RNAi-treated embryos, N2 showed a steep drop in transcript abundance, CB4856 showed no change, and QX1211 showed an on/off pattern with N2-like levels for some, but not most, embryos. This pattern in QX1211 was replicated for a second target, *par-4* (Fig. 2d); see Supplementary File 1 for statistical details. Thus, the patterns of transcript knockdown following RNAi are highly consistent with our prior observations of strain-specific responses.

To examine how transcript abundance, with and without degradation by RNAi, changes with embryonic development, we evaluated embryos with up to 30 nuclei. In the control condition, *par-1* transcripts decreased with embryonic stage (Fig. 2e) (Charles et al. 2021) at a consistent rate across strains (ANCOVA model comparison, *P* = 0.299). This suggests that change in *par-1* abundance does not differ among strains, though variation among wild strains may be subtle and require high sample size to detect (Kaul et al. 2022). In the treatment condition, the strain-specific patterns of transcript degradation persisted without any apparent effect of development on the RNAi response (Fig. 2e). That is, the treated N2 embryos, following a significant knockdown in transcript number (*P* < 0.001), exhibited a flat slope that implies no change in RNAi response with embryo stage. For CB4856 and QX1211, the rate of change by embryo stage was similar between the treatment and control conditions, with marginal (ω^2^ = 0.017, *P* = 0.012) and nonsignificant changes in slope, respectively; this reflects the overall incompetence generally observed for these strains. However, for QX1211, transcript abundance is poorly explained by the linear rate of change in treated embryos (*R*^2^ = 0.16) compared with control embryos (*R*^2^ = 0.53), owing to a subset of early-stage embryos with low counts of *par-1* transcripts (Fig. 2e). We attribute this outcome to an RNAi competent response in a subset of QX1211 hermaphrodite individuals, as we observed in the embryonic lethality experiments (Fig. 1c). (The complete statistical report for this analysis, including estimates of the variance explained and significance levels for ANCOVA model comparisons, is in Supplementary Table 2.) Thus, in this narrow window of embryogenesis and among embryos retrieved from a fixed-age parent, we find no evidence of changing rate of degradation by embryo stage.

In sum, these experiments confirm that the distinct responses of CB4856 and QX1211 are driven by variation in RNAi mechanism, not in developmental variation related to the RNAi target. They also illustrate consistent transcript degradation across the early stages of embryogenesis.

### Reduced PPW-1 function does not universally explain loss of germline RNAi

Given the distinct patterns of germline RNAi incompetence in CB4856 and QX1211, we next sought to evaluate the genetic basis for RNAi failure in these and other low-response strains. First, we considered the role of *ppw-1*. The naturally occurring frameshift mutation in *ppw-1* (Tijsterman et al. 2002) is unique to CB4856 in the *C. elegans* Natural Diversity Resource (CeNDR) database (Cook et al. 2017), but we hypothesized that variation in PPW-1 activity arising from other sources might contribute to variation in germline RNAi among wild strains. To test whether reduction of PPW-1 function is a universal aspect of reduced germline RNAi, we performed complementation tests by crossing N2 wild-type and null alleles of *ppw-1* to 7 wild strains. We evaluated CB4856, QX1211, and 5 additional strains, selected based on evidence of weak germline RNAi in our hands or from prior reports (Paaby et al. 2015) and representation of nucleotide diversity and divergence across the global population (Cook et al. 2017) (see Materials and methods).

We crossed each wild strain to N2 with its native, wild-type copy of *ppw-1* and also to N2 carrying the *ppw-1* deletion allele (*pk1425*). Two genetic incompatibilities segregating within *C*. *elegans* (Seidel et al. 2008, 2011; Ben-David et al. 2017) complicated our crosses, one of which we controlled with a knockout allele at *peel-1*; details are provided in Supplementary File 2. For each cross, we compared the response of the individual wild strain to the 2 heterozygote genotypes in the F1 generation, with and without the deletion at *ppw-1* inherited from the N2 chromosome. We reasoned: though N2 homozygous for the *ppw-1* deletion fails to exhibit an RNAi response, one copy of wild-type *ppw-1* fully rescues it, indicating that *ppw-1* is haplo-sufficient, at least in the N2 background (Fig. 3a); therefore, if weak RNAi in the wild strains is a consequence of reduced PPW-1 activity, any restoration of response in the F1 genotypes should be greater in the genotype with the functional N2  *ppw-1* allele. As previously, we induced *par-1* RNAi in the (F1) parent germline and measured embryonic lethality in the following generation. To avoid confounding differences in developmental timing with variation in RNAi response, we scored all progeny from only the first 15 h of egg-laying from a small pool of hermaphrodite parents (∼100–200 embryos) on each replicate plate.

The 7 strains exhibited 4 distinct response patterns: (1) no rescue, (2) *ppw-1*-dependent rescue, (3) *ppw-1*-dependent suppression, and (4) *ppw-1*-independent rescue, described in detail below. These results indicate that within *C. elegans*, PPW-1 activity varies, PPW-1 activity differentially affects germline RNAi due to interaction with other varying factors, or both. The results further suggest that weak germline RNAi is multigenic within each strain, since rescued responses were all lower than N2 levels, indicating the presence of factors other than *ppw-1*.

1. Strain JU1522 showed no rescue, i.e. no improved RNAi response in either F1 genotype (Fig. 3b). This suggests that weak RNAi in JU1522 is independent of *ppw-1* or at least that alleles that promote RNAi in N2, including *ppw-1*, are not haplo-sufficient to increase the response in the JU1522 background.
2. Strains CB4856 and DL238 exhibited *ppw-1*-dependent rescue: an increased RNAi response when crossed to N2 but only in the background with the wild-type *ppw-1* allele (Fig. 3c, Supplementary Fig. 2). This outcome in CB4856 is consistent with prior reports (Tijsterman et al. 2002; Pollard and Rockman 2013). In DL238, replicate-to-replicate variation in embryonic lethality was high, so we investigated whether this could be explained by potentially stochastic induction of the RNAi response between individual worms. This appears to be the case: tested individually, some hermaphrodites produced no dead embryos and others over 30% (Fig. 3c).
3. Unexpectedly, QX1211 showed *ppw-1*-dependent suppression: the heterozygote with the *ppw-1* deletion allele exhibited a significant increase in embryonic lethality, implying that reduction of *ppw-1* in this strain promotes germline RNAi (Fig. 3d). As expected in this cross, we also observed lethality arising from the genetic incompatibility at the *sup-35;pha-1* locus (Ben-David et al. 2017) (details in Supplementary File 2).
4. Strains CB4852, KR314, and ECA369 exhibited *ppw-1*-independent rescue, in which the 2 heterozygote genotypes exhibited levels of embryonic lethality that were equivalent to each other and significantly higher than the wild strain on its own. This suggests that N2 alleles other than *ppw-1* promote the RNAi response in these genetic backgrounds (Fig. 3e).

To ensure that differences in lethality came from variation in RNAi genes and not from developmental variation specific to *par-1* (Paaby et al. 2015), we introgressed a germline-expressed GFP construct into 4 strains representing the 4 observed response patterns and quantified fluorescence following RNAi against GFP. In contrast to N2, which showed significantly lower fluorescence in treated individuals at all timepoints, CB4856, QX1211, and JU1522 showed no response at the first timepoint, in young adulthood (Supplementary Fig. 3). At later ages, and similar to the embryonic lethality outcomes (Fig. 1c), CB4856 and QX1211 showed increasing responses, while JU1522 showed no significant response at any timepoint; this strain exhibits the weakest response we have observed, including no rescue in the N2 background (Fig. 3b). With the exception of ECA369, which showed higher than expected RNAi sensitivity, the responses are consistent with our gene-specific estimates of RNAi competency.

The results of these complementation tests implicate functional variation at genes other than (or in addition to) *ppw-1*, indicating that wild strains likely carry mutations affecting RNAi at multiple genes. Moreover, they demonstrate diversity in the function or effect of PPW-1 activity on the RNAi response within *C. elegans*. However, with the exception of the unique *ppw-1* frameshift in CB4856, the extent to which RNAi alleles are likely to be strain-specific, vs shared across the population, is unclear.

### Genetic complementation between wild strains implicates diverse and polygenic basis for germline RNAi incompetence

To examine whether alleles limiting germline RNAi are shared across strains, we crossed low-response strains to each other and looked for complementation. As above, we measured embryonic lethality following *par-1* RNAi exposure in the F1 generation. We performed a total of 6 tests, with each pairwise cross for strains CB4856, DL238, KR314, and QX1211 (Fig. 4), chosen based on their diversity in PPW-1 function (Fig. 3) and compatibility at the *zeel-1*;*peel-1* locus (Andersen et al. 2012) (see Supplementary File 2 for more details).

We observed multiple instances of complementation, indicating that variation in RNAi is polygenic and that low-response strains carry alleles with distinct functional effects. For example, in the CB4856 × QX1211 cross, the heterozygote produced significantly more dead embryos than either strain on its own (Fig. 4a), indicating that alleles that dampen the RNAi response are not shared since their function is at least partially rescued by the alternate genetic background. Given that CB4856 and QX1211 exhibit responses that are dependent on *ppw-1* but opposite to each other (Fig. 3), their complementation may be occurring at *ppw-1* itself. However, despite similar responses under *ppw-1* manipulation (Fig. 3), DL238 and CB4856 also complement (Fig. 4b), suggesting distinct mechanisms. DL238 failed to complement QX1211 (Fig. 4c), but did complement KR314 (Fig. 4d), indicating shared and distinct mechanisms, respectively; these outcomes are the opposite of those observed for CB4856 crossed to the same strains (Fig. 4a and e), reinforcing the conclusion that CB4856 and DL238 harbor distinct genetic mechanisms. We also saw evidence for distinct mechanisms in the complementation of KR314 × QX1211 (Fig. 4f).

The rescued responses of the crossed strains indicate diversity in the genetic mechanisms that underlie germline RNAi response in *C. elegans*. Together, the 2 sets of complementation assays (Figs. 3 and 4) demonstrate that RNAi incompetence is multigenic within individual strains and caused by diverse alleles with distinct functional effects. This in turn indicates that variation in RNAi is a polygenic phenomenon within *C. elegans* and suggests that it may be mediated by rare variants. In addition to *ppw-1*, causal mutations may reside in other Argonautes: though *ppw-1* is essential for germline RNAi in N2, overexpression of other WAGOs can rescue the response, implicating interchangeability (Yigit et al. 2006). We hypothesize that natural variation in the expression or function of RNAi genes, specifically WAGOs, produces a phenomenon of gene regulation by small RNAs that is highly diversified within the species. Variability in multiple factors is consistent with the dramatic range in sensitivity to germline RNAi overall (Paaby et al. 2015) as well as the diversity in genetic mechanisms underlying incompetence observed here.

### *C. elegans* RNAi genes show unusually high variation in expression

To assess whether and how RNAi genes might vary in expression across strains, we performed RNA-seq on low-response strains CB4856 and QX1211, on responsive strains N2 and JU1088, and on strain EG4348, which shows an intermediate response (Paaby et al. 2015). RNA samples were prepared from young, reproductively mature hermaphrodites without RNAi induction. Relative to other genes in the genome, RNAi genes, in particular Argonautes, showed highly elevated expression variation across strains, including *ppw-1* and the related WAGO *sago-2*.

**Fig. 5.**
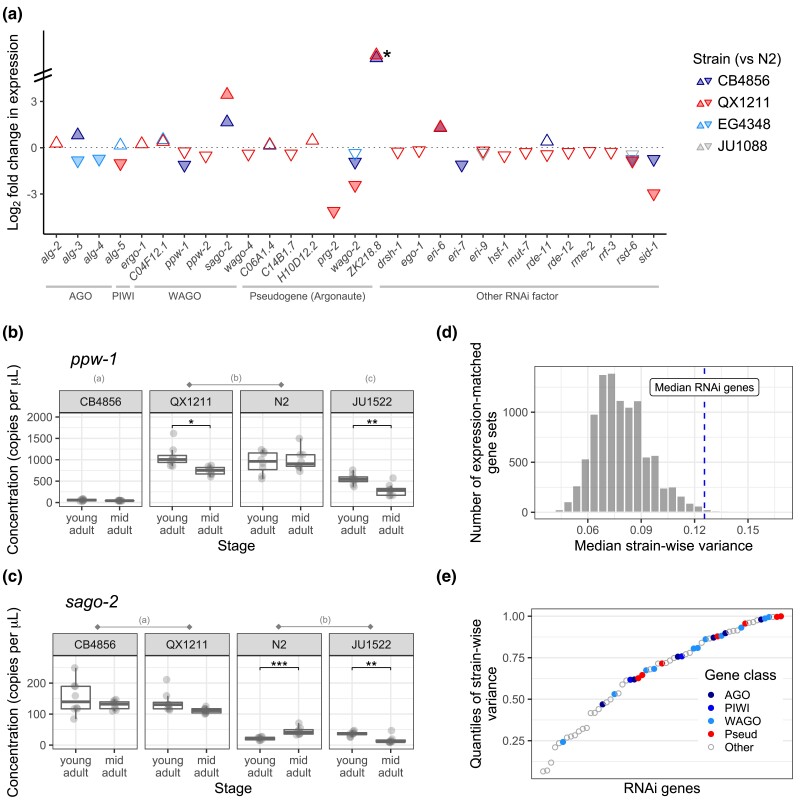
Variation in gene expression for RNAi factors. a) Via RNA-seq, we examined 62 genes for differential expression between N2 and germline RNAi incompetent strains CB4856 and QX1211, highly competent strain JU1088, and moderately competent strain EG4348. Only genes with significant results (FDR < 0.1) are displayed; filled arrows indicate fold change >1.5. The (*) at *ZK218.8* indicates differential expression beyond the *y*-axis scale; this gene is not expressed in the N2 reference strain and has been classified as a pseudogene. b–c) Expression differences via droplet digital PCR for *ppw-1* and *sago-2*. Ten strains were evaluated (Supplementary Fig. 4); a subset is shown here. Across strains, significant differences (Tukey's contrasts, *P* < 0.05) are indicated by letter groupings; for example, QX1211and N2 have equivalent concentrations of *ppw-1*, while CB4856 and JU1522 concentrations are significantly different from all others. Within each strain, significant differences between developmental stages (pairwise contrasts with Bonferroni correction) are indicated by *P* < 0.001 (***), *P* < 0.01 (**), and *P* < 0.05 (*). d) Histogram of median strain-wise variance for 10,000 gene sets, expression-matched to the RNAi genes, randomly sampled across the genome following variance-stabilizing transformation. e) Strain-wise variance of the 61 expressed RNAi genes, plotted by quantile of genome-wide variances (gene expression data as in (d)). Argonautes, including those currently classified as pseudogenes, are indicated by filled circles.

First, we examined expression at 62 genes known to directly mediate RNAi (listed in Table 1), including Argonautes currently classified as pseudogenes on WormBase (Harris et al. 2020). Of these, all but 2 were expressed in every strain: *wago-5* was not expressed at all, and the putative pseudogene *ZK218.8* was not expressed in the responsive stains but was expressed in CB4856 and QX1211. Of the genes with detectable transcripts, approximately half (29/61) exhibited differential expression (FDR < 0.1) between N2 and at least one other wild strain (Fig. 5a).

**Table 1.** Molecular diversity at 62 RNAi genes, from 403 strain isotypes.

Class	Gene	Nucleotide diversity (π)	No. strains with functional diverged alleles	No. strains with pseudo (missing) alleles	Class	Gene	Nucleotide diversity (π)	No. strains with functional diverged alleles	No. strains with pseudo (missing) alleles
Argonaute (AGO)	*alg-1*	7.20E−04	0	0	Other RNAi factors	*eri-1*	1.23E−03	23	0
	alg-2*	8.53E−04	0	19		*eri-12*	1.26E−03	23	0
	*alg-3*	6.86E−04	0	1		*eri-3*	9.37E−04	9	0
	*alg-4*	6.08E−04	10	5		*eri-5*	9.64E−06	0	1
	*alg-5*	3.75E−04	0	0		*eri-6*	7.70E−06	36	20
	*rde-1*	6.04E−04	2	0		*eri-7*	9.17E−06	0	12
Argonaute (PIWI)	ergo-1*	5.35E−04	0	8		*eri-9*	1.07E−03	26	0
	*prg-1*	4.37E−04	0	0		*hrde-2*	6.59E−04	0	0
Argonaute (WAGO)	*C04F12.1*	4.98E−04	0	34		*hsf-1*	7.58E−05	106	0
	*csr-1*	2.50E−04	0	0		*lin-15b*	4.08E−04	9	53
	*hrde-1*	3.00E−04	4	3		*mut-7*	6.37E−04	0	0
	*nrde-3*	1.11E−03	22	0		*nck-1*	2.35E−04	0	0
	*ppw-1*	2.37E−04	0	2		*nhl-2*	1.07E−04	0	0
	*ppw-2*	5.00E−04	0	0		*nyn-2*	1.74E−04	2	1
	*sago-1*	7.21E−04	0	4		*rde-10*	7.12E−04	21	1
	*sago-2*	5.03E−06	1	25 (3)		*rde-11*	9.92E−04	0	1
	*wago-1*	3.06E−04	0	0		*rde-12*	2.61E−04	54	0
	wago-10*	9.03E−04	58	2		*rde-2*	8.90E−04	0	0
	*wago-4*	1.23E−03	0	0		*rde-4*	5.01E−04	0	0
	*wago-5*	6.59E−04	0	48		*rde-8*	2.22E−04	0	0
Argonaute (pseudo)	*C06A1.4*	1.18E−03	0	0		*rme-2*	4.47E−04	0	0
	*C14B1.7*	1.77E−03	0	0		*rrf-1*	1.00E−03	9	0
	*H10D12.2*	6.47E−04	0	25		*rrf-3*	5.45E−05	14	0
	*prg-2*	7.15E−04	0	3 (1)		rsd-2*	2.46E−03	151	4
	*wago-11*	1.44E−03	0	64		rsd-3*	1.48E−04	35	0
	*wago-2*	2.09E−04	0	0		*rsd-6*	2.09E−04	28	0
	ZK218.8*	5.55E−03	0	11 (10)		*sid-1*	1.09E−03	0	1
Other RNAi factors	*dcr-1*	6.59E−04	49	0		*sid-2*	1.19E−03	54	3
	drh-1*	1.84E−04	40	0		*sid-3*	2.67E−03	56	1
	*drsh-1*	3.39E−04	0	0		*sid-5*	2.00E−04	0	0
	*ego-1*	2.77E−04	0	0		*tofu-5*	1.23E−03	0	0

Genes were manually curated from the literature and include 20 putatively functional Argonautes and 7 currently classified as pseudogenes on WormBase (Harris et al. 2020), though pseudogene status varies in the literature (and likely across strains). “Functional diverged alleles” are those with at least 1% nucleotide divergence from the reference genome, including at least 5 moderate mutations, such as amino acid substitutions, and no disruptive high-impact mutations, such as frameshifts or stop-gains. “Pseudogenized alleles” are those with at least one high-impact mutation called with high confidence and at which 1% or more of the sites are diverged or missing or those with at least 50% of the sites with missing calls. Cases with more than 75% of sites uncalled may more likely represent missing genes and are indicated in parentheses. Potential pseudogenization was assessed with respect to the reference genome and considered even for those Argonautes already classified as putative pseudogenes. Genes located in an interval of hyperdiversity (Lee et al. 2021) in at least one strain are indicated by an asterisk.

The strains with greatest differential expression were those with weakest germline RNAi (Fig. 5a). That is: QX1211, then CB4856, showed the most differences across the gene set (26/61 and 10/61), the moderately responsive strain EG4348 showed a handful of differences (7/61), and the highly responsive strain JU1088 showed a difference at only one gene (*rsd-6*) (Fig. 5a). Of genes differentially expressed by both CB4856 and QX1211, the direction of expression was concordant with one exception (*rde-11*), including reduced expression of *ppw-1* and elevated expression of *sago-2*. It is critical to note that though QX1211 and CB4856 show the greatest degree of differential expression relative to N2, they are also the most genetically diverged (Cook et al. 2017). That said, RNAi incompetence does not appear to be a function of genetic distance from the reference strain, as highly diverged isolates ECA701, JU561, and XZ1516 (Crombie et al. 2019) were responsive to *par-1* RNAi (Supplementary Fig. 5) and RNAi sensitivity for 55 wild strains in Paaby et al. (2015) showed no relationship with divergence from N2, either genome-wide or for RNAi genes specifically (Supplementary Fig. 6).

The WAGO *sago-2* shares high sequence identity with *ppw-1* and resides ∼17 cM away on chromosome I. These 2 genes share overlapping function in the N2 background (Yigit et al. 2006), so the underexpression of *ppw-1* and overexpression of *sago-2* in CB4856 and QX1211 (Fig. 5a) caught our attention. However, in some strains, including QX1211, poor mapping of short reads to the reference genome (Cook et al. 2017) at these loci suggests gene divergence or duplication. We resolved sequence ambiguities via de novo assembly of paired-end reads and long-read sequencing and observed that QX1211 carries *ppw-1*-like alleles at both loci (Supplementary File 3). Therefore, to confirm our RNA-seq observations of *ppw-1* and *sago-2* expression and also to evaluate additional strains, we designed a droplet digital PCR (ddPCR) experiment to measure both transcripts simultaneously and discriminate between them using transcript-specific labels. We tested all strains thus far discussed: the 7 low-response strains for which we tested *ppw-1* function (Fig. 3), as well as responsive strains N2, JU1088, and EG4348. To evaluate whether *ppw-1* or *sago-2* expression changed as worms aged, we also assayed 2 developmental timepoints, young adult and mid-adult.

The ddPCR results were consistent with our RNA-seq observations and in sum confirm high variability in *ppw-1* and *sago-2* expression (Fig. 5b and c, Supplementary Fig. 4). Overall, *ppw-1* expression was about an order of magnitude greater than that of *sago-2*, and both expression levels and changes in expression between developmental timepoints differed significantly across strains for both genes (Supplementary Table 3). Taken individually, neither *ppw-1* nor *sago-2* expression correlated with RNAi responsiveness, and across all 10 strains, the combined expression was both highest and lowest in 2 strongly resistant strains: QX1211 and JU1522, respectively (Fig. 5b and c, Supplementary Fig. 4). JU1522 has consistently exhibited negligible germline RNAi, including no rescue when crossed with N2 (Fig. 3) and no response even at later age (Supplementary Fig. 3). One possibility is that both high and low expression of *ppw-1* and *sago-2* limit germline RNAi. This hypothesis fits with our observation that a haploid dose of *ppw-1* increases the RNAi response in QX1211 (Fig. 3d) and with the prior finding that *ppw-1* and *sago-2* encode functionally interchangeable proteins that can compensate each other (Yigit et al. 2006). However, it is inconsistent with the observation that overexpression of these factors increases RNAi sensitivity in N2 (Yigit et al. 2006). N2 and JU1088, the 2 strains with the most robust germline RNAi response in our analysis, exhibited intermediate levels of *ppw-1* and *sago-2* combined (Supplementary Fig. 4). We also note that while *ppw-1* expression is not significantly different between QX1211 and N2 summed across developmental timepoints (Fig. 5a), expression in QX1211 is lower at the mid-adult stage (Mann–Whitney U test, *W* = 55, *P* = 0.0074), consistent with the RNA-seq results (Fig. 5a).

Having observed significant expression variation for many of the RNAi genes, we next asked whether this gene set is more variable than other genes in the genome. The answer is yes: for the 61 actively transcribed RNAi genes, the median strain-wise variance (after variance-stabilizing transformation, see Materials and methods) was higher than that of expression-matched sets randomly sampled from the genome 99% of the time (9,936/10,000 comparisons; Fig. 5d); 74% of these (7,351/10,000) were statistically significant (one-tailed Mann–Whitney test, alpha = 0.05), far exceeding that expected by chance. This effect is driven by elevated variance across the gene set, not by a few outliers of high variance: the majority (57%) (35/61) exhibit significant differences by strain via LRT (FDR < 0.1) compared with 29% of all genes in the genome, and the proportion of differentially expressed genes was higher than the random sample 99.9% of the time (9,991/10,000 comparisons, *P* = 9 × 10^−4^ by permutation). Thus, while many genes are differentially expressed across the genome (Supplementary Fig. 7), RNAi genes are highly enriched for strain-wise variation (*P* = 4.8 × 10^−6^, hypergeometric test).

Several of the RNAi genes are exceptionally variable. The putatively pseudogenized Argonaute *prg-2* exhibits the 10th highest strain-wise variance in the genome, and 6 genes, including *ppw-1* and *sago-2*, are in the top 2%. Given the relatively recent evolution of WAGOs (Buck and Blaxter 2013) and their potential redundancy in function (Yigit et al. 2006), we expected to see especially high expression variation for this gene class. This hypothesis is well supported: all but one (*sago-1*) of the 12 WAGOs are in the top half of genome-wide variance, though Argonautes of all classes showed a similar trend, including those presumed to be pseudogenes (Fig. 5e). The distribution of strain-wise variances was more evenly distributed for other RNAi factors and included both highly variable and highly invariant expression patterns (Fig. 5e).

The elevated expression variation in RNAi genes represents variation in small RNA processes in *C. elegans*, consistent with the other evidence for diversification of germline RNAi function within the species and a possible explanation for that diversity. Variable expression of putative pseudogenes, including the active transcription of *ZK218.8* in some nonreference strains, also prompts the question of whether wild strains vary in their complement of functional Argonautes. *ZK218.8* was previously identified as an Argonaute (Yigit 2007) but remains unexplored in the literature; its expression signature suggests that pseudogenization may have occurred in some strain lineages but not in others. While these observations all point to diversification in genetic mechanisms of RNAi, the historical forces driving these outcomes remain obscured. To evaluate this, and to look for evidence of strain-specific mutations affecting RNAi, we next turned to population-level sequence data.

### *C. elegans* RNAi genes show lineage-specific diversification and pseudogenization

To identify candidate mutations for RNAi incompetence in the 7 low-response strains we evaluated, as well as to assess historical selection on RNAi processes within *C. elegans* generally, we examined allelic diversity at the 62 RNAi genes in CeNDR, which includes hundreds of strains representing the global *C. elegans* population (Cook et al. 2017). For the 7 low-response strains, we identified putatively deleterious variants that may contribute to individualized loss of function, as many were strain-specific (Supplementary Table 4). Among these 7 strains, none of the 62 RNAi genes fall in an interval designated as hyperdiverse (Lee et al. 2021), with the exception of *ergo-1* in ECA369. Across the population, we observed substantial sequence variation among all gene classes, including strain-specific instances of pseudogenization and allelic divergence (Table 1).

**Fig. 6.**
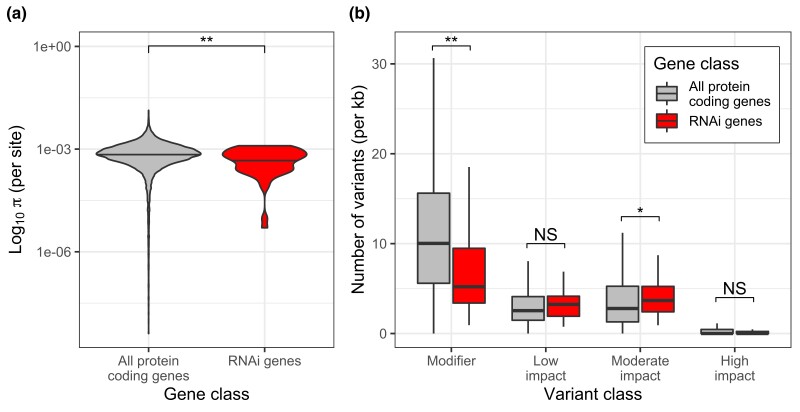
Comparison of mutational variation in RNAi genes vs all genes. a) Per-site nucleotide diversity and b) variant counts, per kb, for multiple impact classes. Results shown here excluded hyperdiverged regions (Lee et al. 2021); pseudogenes are also excluded from the variant counts, as they affect impact classification. Significance levels (2-tailed Mann–Whitney U): *P* < 0.01 (**) and *P* < 0.05 (*).

Relative to all protein-coding genes in the genome, RNAi genes exhibit lower nucleotide diversity (median π per site for all genes = 6.96E−4; median for RNAi genes = 6.06E−4; *P* = 0.022 by Mann–Whitney U) (Fig. 6a); this is true whether or not genes in hyperdiverged regions (Lee et al. 2021) are included in the analysis (Supplementary Table 5). However, RNAi genes are enriched for higher impact variants, including those predicted to change the amino acid sequence (Fig. 6b). Elevated variation was often, but not always, associated with high-impact mutations in one or more strains (Supplementary Fig. 8). Therefore, to distinguish between instances of pseudogenization and gene loss vs functional allelic diversification arising from directional or balancing selection, we classified highly diverged alleles 2 ways. We considered an allele to be pseudogenized if it harbored at least one high-impact mutation called with high confidence and if at least 1% of the sites were diverged or missing relative to the reference genome or if over 50% of sites were missed calls; we classified “functional diverged alleles” as those with at least 5 amino acid substitutions, at least 1% divergence across sites, and no high-impact mutations called at the locus.

**Fig. 7.**
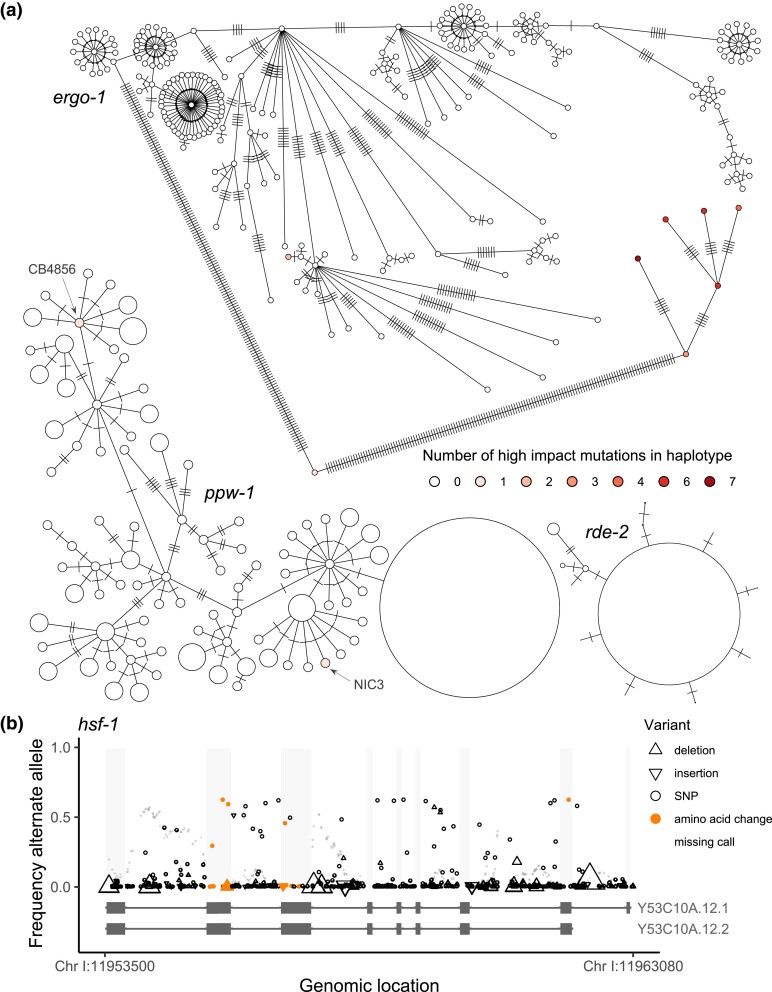
Population-level allelic diversity in RNAi genes across 403 strains. a) The WAGO Argonaute *ppw-1* exhibits relatively elevated polymorphism, and 2 strains carry independent high-impact mutations: the frameshift in CB4856 and a stop-gain in NIC3. The PIWI Argonaute *ergo-1* exhibits extreme polymorphism, including accumulation of multiple high-impact mutations in some alleles. In contrast, the RNAi-deficient gene *rde-2* exhibits conservation and no putatively loss-of-function mutations. In these networks, each circle represents a unique haplotype (of one or more strains) and hatch marks indicate mutations; for *ppw-1* and *rde-2*, haplotype circles are scaled by frequency. b) The transcription factor *hsf-1* also shows elevated polymorphism. Multiple changes to the amino acid sequence, mutations segregating at intermediate frequency, and no observed high-impact mutations that disrupt the protein suggest functional divergence. Each observed mutation is represented as a single point; the up and down triangles representing indels are scaled by indel length (range = 1–99, mean = 6.8).

We observed extensive pseudogenization across the gene set. Given the expansion and diversification of Argonautes in nematodes (Buck and Blaxter 2013), we hypothesized that the WAGOs might be relatively unconstrained and therefore especially susceptible to gene loss. Indeed, many WAGOs (7/13) showed evidence of relaxed selection, including *ppw-1*: in addition to the loss-of-function frameshift mutation in CB4856, the strain NIC3 carries an independent stop-gain (Fig. 7a) at amino acid 599, which is in the PIWI domain. However, putative loss occurred in all gene classes, including the PIWIs, AGOs, and other RNAi factors (Table 1). For example, the PIWI Argonaute *ergo-1* exhibits extensive variation and lineage-specific pseudogenization (Fig. 7a). Excluding the 7 Argonautes classified as pseudogenes on WormBase (Harris et al. 2020), 40% (22/55) of genes indicated pseudogenization in one or more strains (Table 1). Further, the Argonautes *sago-2*, *prg-2*, and *ZK218.8* appear to be missing in some strains (Table 1, Supplementary Fig. 8). In contrast, a few genes exhibited very low polymorphism (Fig. 7a, Supplementary Fig. 8), likely reflecting evolutionary constraint and purifying selection. These genes were mostly non-Argonautes, with the exception of *csr-1* and *prg-1*, the only Argonautes essential for development (Yigit et al. 2006).

We also observed pervasive functional divergence, with functionally diverged alleles in 25/62 genes (Table 1). Unlike the pseudogenized alleles, which often occurred singly, the functional diverged alleles were most often shared across strains, consistent with positive selection driving or maintaining divergent gene function. For example, *hsf-1* exhibited very high polymorphism, including amino acid changes at intermediate frequencies and one of the highest haplotype diversities of all 62 genes (Supplementary Table 6) but no instances of high-impact mutations likely to knock out function (Fig. 7b). (We also observed poor read mapping across the locus, which indicates further divergence but may also obscure deleterious mutations, though *hsf-1* does not fall in an interval of hyperdiversity (Lee et al. 2021).) This transcription factor is a master regulator of other RNAi genes, and HSF-1 activity is associated with transgenerational inheritance of an on/off RNAi response (Houri-Zeevi et al. 2020). As *hsf-1* is a potential keystone regulator of small RNA pathways, its diversification may underlie significant functional variation in RNAi.

The patterns of polymorphism suggest that RNAi genes are evolving dynamically within *C. elegans*, with lineage-specific trajectories of relaxed selection and gene loss, as well as possible directional selection and functional divergence. The occurrence of rare alleles and lineage-specific pattern findings are consistent with our experimental observations that the genetic basis of RNAi failure is strain-specific. Moreover, they suggest that functional characterizations of these genes, which have been universally achieved in N2, may be strain-specific as well.

## Discussion

In this study, we demonstrate that a diversity of genetic mechanisms underpins the failure of some wild *C. elegans* strains to mount a robust germline RNAi response. Rather than identifying one or more common, shared factors that explain RNAi incompetence, our results indicate that RNAi fails for different reasons in different strains and that the same genes can produce opposite responses. Specifically, we show that natural variation at genes other than *ppw-1* contributes to germline RNAi incompetence in wild *C. elegans* and that while loss of PPW-1 function weakens the RNAi response in some strains, it appears to amplify it or have no effect in others. Coupled with high levels of divergence and lineage-specific pseudogenization at known RNAi genes, these findings indicate that the small RNA pathways in *C. elegans* are evolving rapidly and dynamically, leading to functional diversification of RNAi activity.

We propose that such diversification evolved as a consequence of (1) redundancy and interchangeability among Argonautes (Yigit et al. 2006; Billi et al. 2014 May 7), (2) competition between overlapping pathways (Yigit et al. 2006; Youngman and Claycomb 2014), and (3) a population structure with reduced gene flow (Dolgin et al. 2007). Small RNA processes dominate the biology of *C. elegans* (Youngman and Claycomb 2014; Houri-Zeevi et al. 2020), and defenses against pathogens and transposable elements may be especially susceptible to strong selection (Nuez and Félix 2012). However, because the species is globally dispersed and reproduces primarily by selfing, *C. elegans* lineages evolve semi-independently, may be exposed to distinct selection pressures, and may accumulate coadapted allelic combinations (Dolgin et al. 2007; Campbell et al. 2018). Argonautes and other factors are shared among pathways, and competition between exogenous and endogenous RNAi can force induction of one pathway over another (Yigit et al. 2006; Youngman and Claycomb 2014). In this vein, we might imagine, for example, how selection on germline maintenance in one genetic background could compromise a response to environmental triggers, as well as how Argonaute redundancy could facilitate evolutionary lability and gene- and lineage-specific responses even under similar selection pressures. The rapid evolution of RNAi processes across taxa, particularly within nematodes, implicates RNAi as a rich substrate for adaptive response (Winston et al. 2007; Obbard et al. 2009; Nuez and Félix 2012; Buck and Blaxter 2013; Buck and Blaxter 2013).

An example of competition between overlapping pathways may be reflected in some of our results, as simultaneous exogenous and endogenous demands on shared factors may explain the behavior of QX1211. In addition to the on/off responses among individuals (Figs. 1 and 2), we observed inconsistent sensitivity to RNAi among QX1211 samples, including increased embryonic lethality that could be attributable to an active exogenous RNAi response, before we rigorously controlled temperature for this strain. Consecutive generations at warmer temperatures induce the mortal germline phenotype and reproductive extinction in QX1211, which is associated with shifts in piRNA-like pools of small RNAs (Frézal et al. 2018). As such, one possibility is that changes in RNAi activity may be either a cause or a consequence of germline mortality in QX1211 within individual animals, which in turn may explain the differences in sensitivity to exogenous RNAi; future experiments are required to directly test this hypothesis. Competition between pathways may also explain the *ppw-1*-dependent suppression of RNAi in QX1211 (Fig. 3). If *ppw-1* is a limiting factor in the defense against germline mortality in QX1211, then decreasing its availability might downregulate germline protection while simultaneously releasing resources for the competing exogenous pathway. QX1211 exhibited highest expression of *ppw-1* and *sago-2* (Fig. 5) and carries a *ppw-1*-like allele of *sago-2* (Supplementary File 3)—in the most speculative case, this might reflect a history of selection for *increased* germline-associated RNAi response in QX1211, even as laboratory assays for exogenous RNAi reveal apparent incompetence.

Consequently, although incompetence for laboratory-induced RNAi is the explicit focus of this study, we emphasize that the synthetic phenomenon of RNAi by feeding does not necessarily represent processes most relevant in nature. The role of RNAi in the wild remains largely obscured, though some observations offer clues. Exogenous RNAi likely induces responses that evolved for antiviral immunity, as viruses that infect *C. elegans* and other *Caenorhabditis* species have been discovered, notably in isolates with defective RNAi, and antiviral immunity shows a clear association with an active RNAi response (Schott et al. 2005; Wilkins et al. 2005; Yigit et al. 2006; Félix et al. 2011; Sarkies et al. 2013). The overlap between experimental RNAi and antiviral response is incomplete, however, as variation in RNAi sensitivity does not completely correlate with immunity and the systemic and transgenerational properties of RNAi are not observed in viral infection (Félix et al. 2011; Ashe et al. 2013; Ashe et al. 2015). Orsay virus, the only naturally occurring virus known to infect *C. elegans*, invades intestinal cells and is horizontally, but not vertically, transmitted (Félix et al. 2011; Franz et al. 2014), though vertically transmissible viral-like RNAs have been detected in the germlines of wild-caught *Caenorhabditis* isolates (Richaud et al. 2019), suggesting undiscovered host–pathogen dynamics. Endogenous RNAi is likely required for germline maintenance in the wild, as suggested by the observations in QX1211. Hence, the piRNA pathway, which is active in the germline and presumed critical for maintaining genome integrity (Wilson and Doudna 2013; Youngman and Claycomb 2014), may dominate the biology of, or be upregulated more often in, some strains relative to others (Frézal et al. 2018). One possibility is that *ergo-1* gatekeeps RNAi pathway activity differently in different isolates, as *ergo-1*  N2 mutants show enhanced exo-RNAi but reduced endo-RNAi (Yigit et al. 2006) and the *ergo-1* locus exhibits extreme allelic diversification in nature (Table 1 and Fig. 7). Another possibility is that in the wild, RNAi in the germline matters most to future generations. Strains resistant to RNAi upon exposure can show transgenerational sensitivity (Tijsterman et al. 2002), and a growing body of research emphasizes the outsized role of RNAi in transgenerational inheritance (Houri-Zeevi et al. 2020, 2021). Thus, RNAi as we have studied it in the lab provides an oblique view into its role in nature.

*C. elegans* increasingly appears to be dominated by transgenerationally inherited small RNA programs (Houri-Zeevi et al. 2020, 2021) that vary significantly in nature (Frézal et al. 2018). This variation offers leverage: characterization of natural variation can elucidate condition-dependent mechanisms (Chandler et al. 2013), which are rampant in RNAi, a complex and intricate collection of interactions susceptible to unexpected outcomes (De-Souza et al. 2019) and sensitive to environmental conditions (Houri-Zeevi et al. 2021). Identifying mechanisms of variation will help to bridge the gulf between our understanding of the genetics of RNAi and the role of RNAi in nature, and future work may benefit from evaluating wild strains in the context of carefully chosen environmental perturbations (Rockman 2008). For example, temperature likely matters for RNAi, given the exquisite sensitivity of *C. elegans* to temperature (Testa et al. 2020) and the intimate relationship between temperature and other stresses and RNAi (Frézal et al. 2018; Houri-Zeevi et al. 2021; Pagliuso et al. 2021); our observations of QX1211 would have been obscured without rigorous temperature control. Sydney Brenner's selection of *C. elegans* as a model species, and N2 as the strain of study, was fortuitous for the future discovery of RNAi (Félix 2008), and it remains the most fertile area for elucidating gene regulation by small RNAs (Youngman and Claycomb 2014). Now, characterizations of significant natural genetic and functional variation in RNAi provide a new access point for expanding our understanding in a system already so well established.

## Supplementary Material

iyad191_Supplementary_Data

## Data Availability

Strains and plasmids are available upon request. All experimental data are provided in Supplementary File 4. DNA sequencing data are available from the NCBI Sequence Read Archive via projects PRJNA1025857 (assembled PacBio data for 2 strains) and PRJNA1025861 (raw Illumina short reads for 9 strains). RNA-seq data and analysis scripts are available via a companion manuscript (Bell et al. 2023) reporting genome-wide gene expression patterns in the 5 strains for which we describe gene expression at RNAi genes. RNA-seq data are also available for gene-specific queries via https://wildworm.biosci.gatech.edu/rnai/.
