# Supplementary material for: Diversification of small RNA pathways underlies germline RNA interference incompetence in wild Caenorhabditis elegans strains
Source: Genetics. 2023 Oct 22;226(1):iyad191. doi: 10.1093/genetics/iyad191 (PMC10763538; doi:10.1093/genetics/iyad191)
Supplement: iyad191_Supplementary_Data [file iyad191_supplementary_data.zip › Main_Supplement_GENETICS-2023-306148.pdf]

SUPPLEMENT CONTENTS

Figure S1. Average brood sizes in *par-1* RNAi experiment over whole lifespan

Figure S2. Complementation test for DL238 with pooled hermaphrodites

Figure S3. RNAi against germline-expressed GFP in wild strains

Figure S4. Droplet digital PCR results for *ppw-1* and *sago-2* for all ten tested strains

Figure S5. Embryonic lethality following RNAi against *par-1* in ECA701, JU561, and XZ1516

Figure S6. RNAi sensitivity for 55 wild strains versus genetic distance from reference strain N2

Figure S7. Genome-wide expression for the reference strain N2 and four wild strains

Figure S8. Population-level sequence variation for 62 RNAi genes

Figure S9. Seven low-response strains chosen for *ppw-1* complementation tests (see separate file)

Figure S10. Embryonic lethality following RNAi against *par-1* in the *ppw-1;peel-2* mutant

Table S1. Statistical results for RNAi in individual worms over lifespan (low-response strains)

Table S2. Statistical estimates for changes in smFISH transcript abundance

Table S3. Statistical results for the ddPCR analysis

Table S4. Candidate genes for weak germline RNAi in the seven strains tested

Table S5. Estimates of nucleotide diversity

Table S6. Haplotype diversity metrics for RNAi genes

Table S7. Strains used in this study

File S1. Statistical details of smFISH in early stage embryos (text below)

File S2. Details of genetic incompatibilities (text below)

File S3. Sequences of *ppw-1* and *sago-2* in CB4856, N2 and QX1211 (see separate file)

File S4. Experimental data (see separate file)

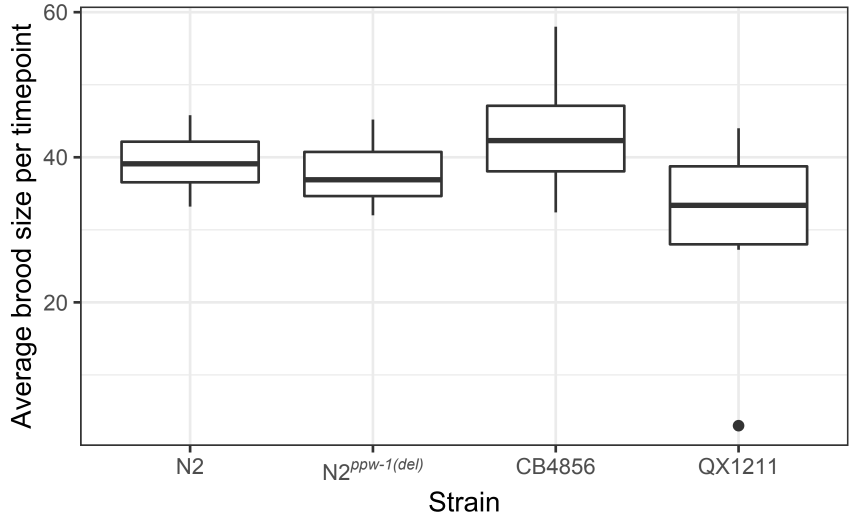

Figure S1. Average brood sizes, per timepoint, for worms in *par-1* RNAi experiment over lifespan.

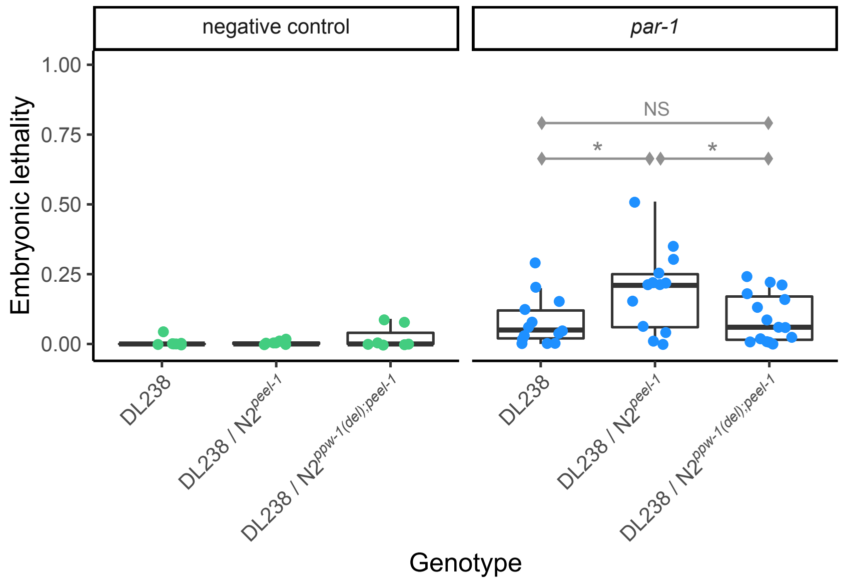

Figure S2. Complementation test for DL238 with pooled hermaphrodites. DL238 was crossed to the RNAi-sensitive laboratory strain N2, with and without a deletion allele at *ppw-1*. The embryos scored here were laid by pooled hermaphrodites on replicate plates. Significance level (Tukey's contrasts): $p<0.05$ (*).

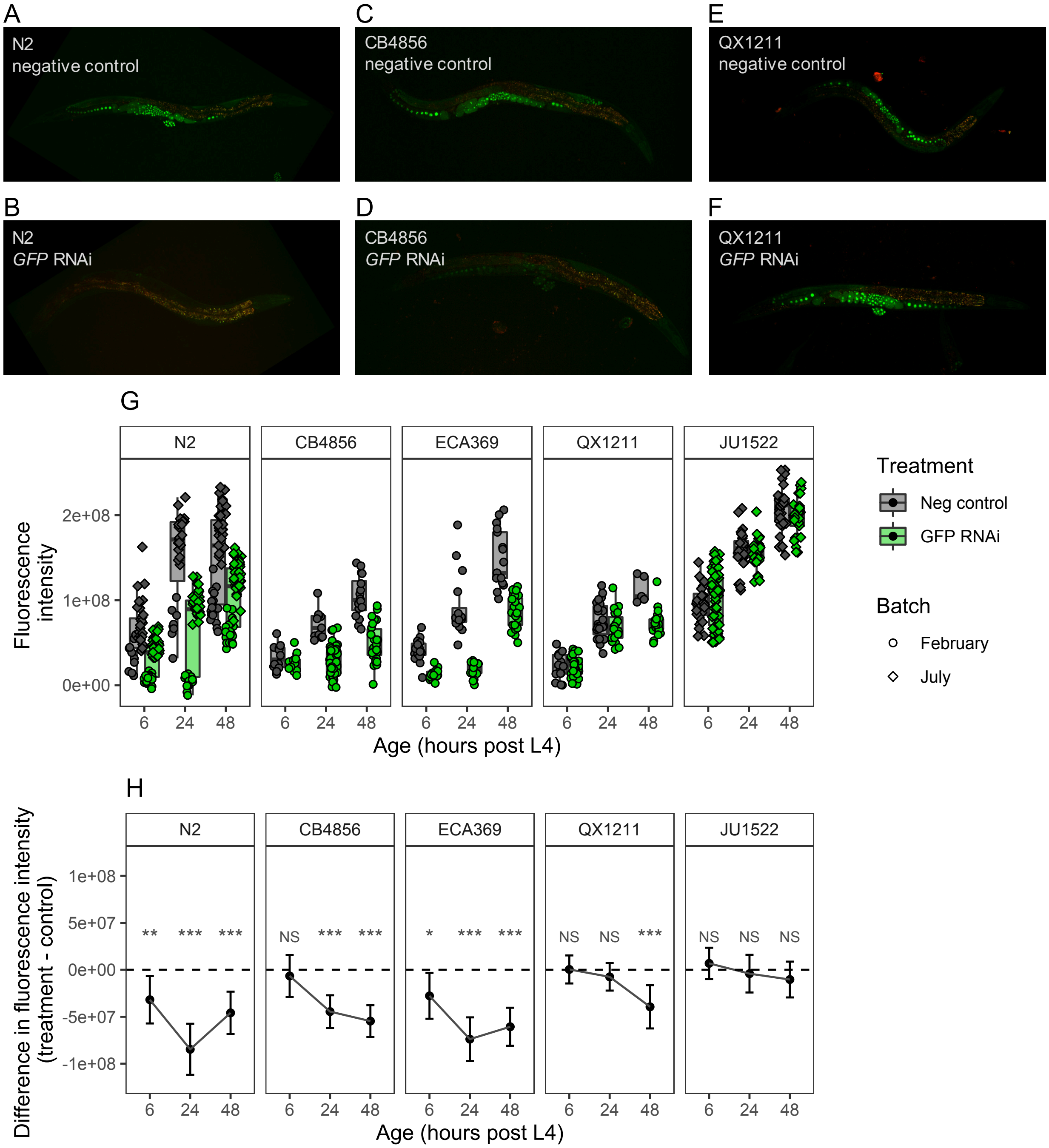

Figure S3. RNAi against germline-expressed GFP in wild strains. (A) - (F) Representative images of control and treated worms, all at 24hrs post L4 stage, are shown for N2, CB4856 and QX121. Green indicates GFP fluorescence; the red channel is overlaid on these images to show autofluorescence. (G) Whole-worm fluorescence intensity for worms imaged at 6, 24, and 48hrs post L4 stage; each point represents fluorescence measured for a single individual. (H) The difference in fluorescence intensity between treated and untreated samples; error bars represent standard error. Significance levels (Tukey's contrasts): $p<0.001$ (***), $p<0.01$ (**), $p<0.05$ (*).

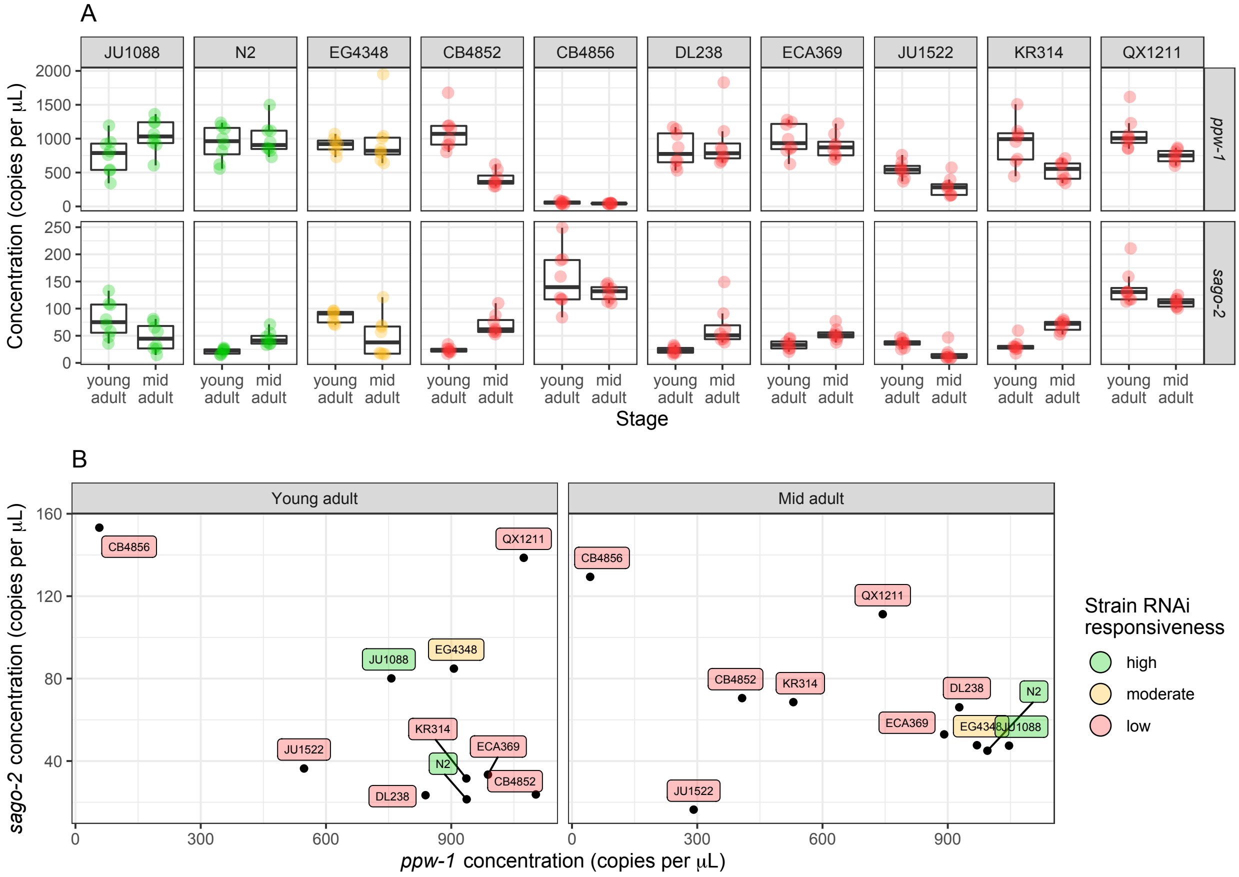

Figure S4. Droplet digital PCR results for *ppw-1* and *sago-2* for all ten tested strains. (A) Strains are ordered by high- (JU1088 and N2), moderate- (EG4348) and low- (remaining strains) responsiveness to RNAi. (B) *ppw-1* vs *sago-2* expression within strains.

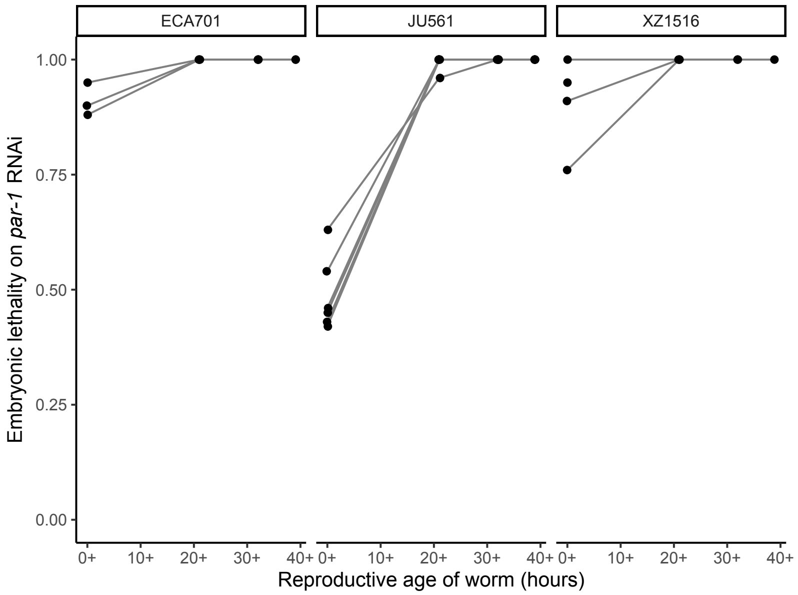

Figure S5. Embryonic lethality following RNAi against *par-1* in wild strains ECA701, JU561, and XZ1516.

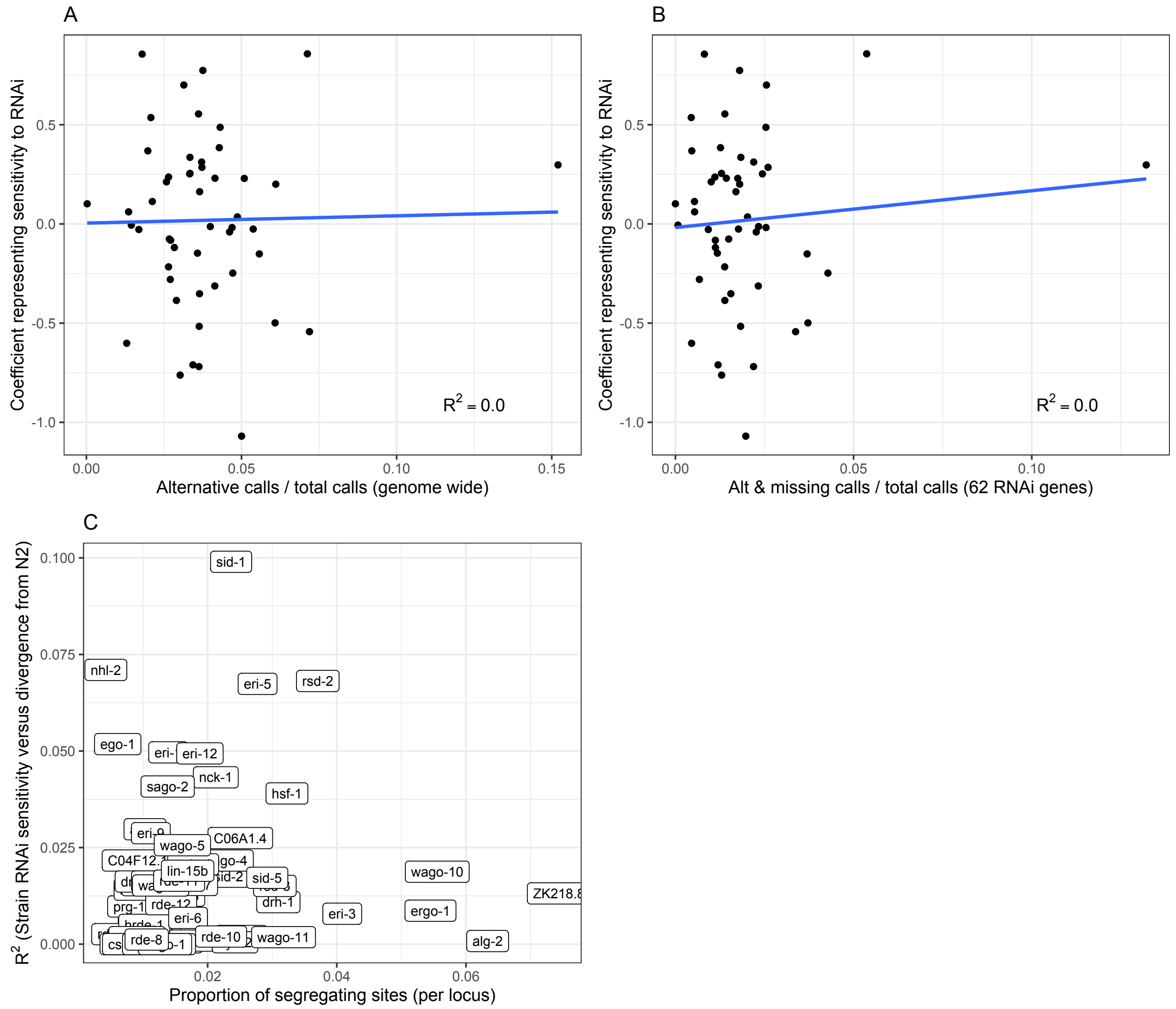

Figure S6. RNAi sensitivity for 55 wild strains versus genetic distance from reference strain N2. RNAi data is from (*Paaby et al., 2015*), which measured embryonic lethality following RNAi by feeding against 29 individual maternal-effect targets; coefficients associated with the strain term in the full statistical model represent strain-specific variation in embryonic lethality associated with RNAi responsiveness, such that increasingly positive values indicate weaker germline RNAi. The coefficients representing RNAi sensitivity for each strain are plotted against strain divergence from N2 (A) genome-wide and (B) averaged over the 62 RNAi genes, using variant data from the CeNDR database (*Cook et al., 2017*). The coefficients were also regressed onto divergence for each RNAi gene individually; (C) shows the R^2 values, plotted against the proportion of segregating sites for each gene.

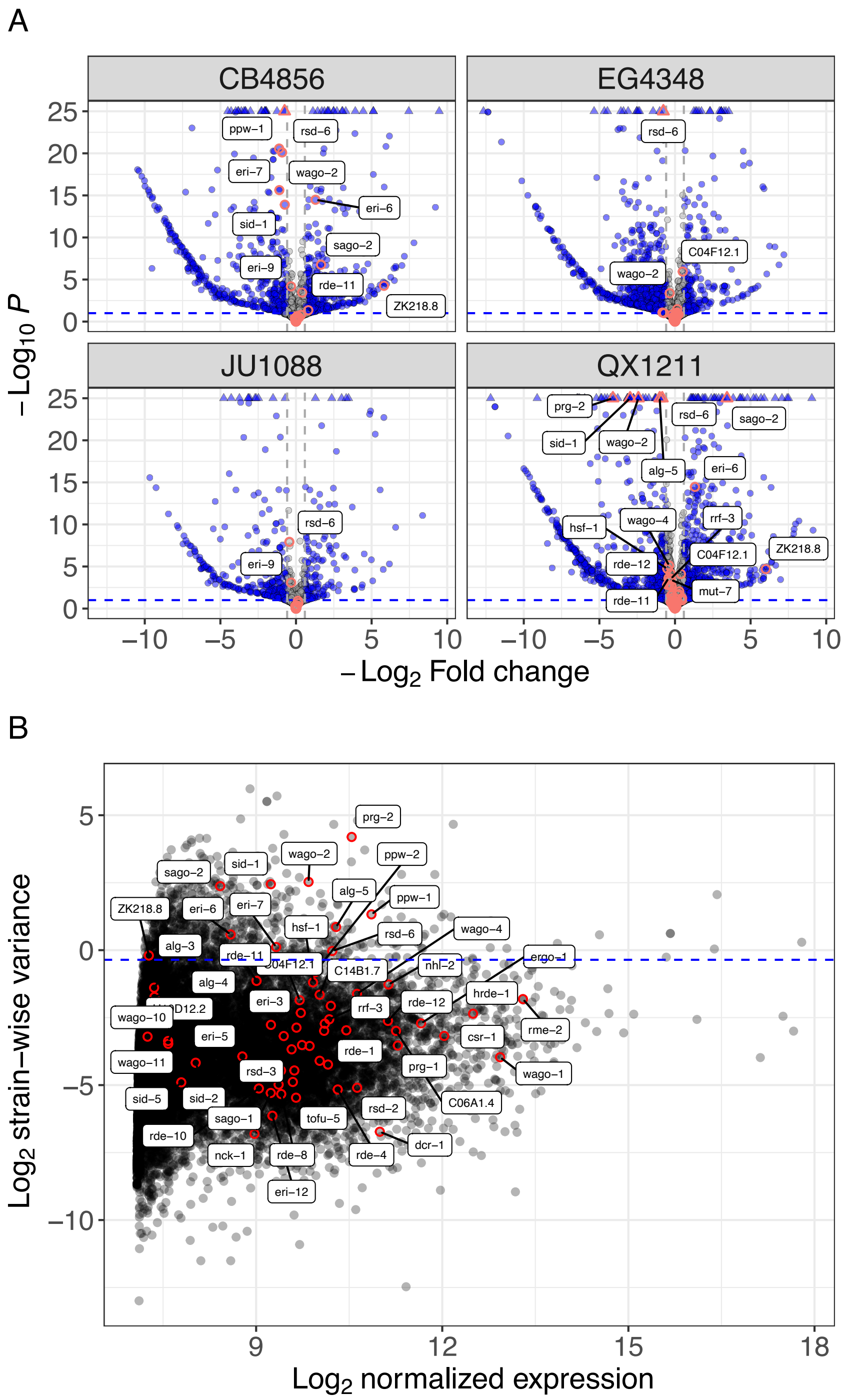

Figure S7. Genome-wide expression for the reference strain N2 and wild strains CB4856 (low RNAi response), EG4348 (moderate response), JU1088 (high response) and QX1211 (low response). RNAi genes are highlighted in red. (A) Volcano plots representing differential expression between N2 and each wild strain. Log2 fold changes are *ashr*-shrunken. Triangles indicate p-values $<1x10^{-25}$. (B) Strain-wise variance in gene expression, represented by ANOVA sum-of-squares (see Methods), plotted against mean expression, which has been variance-stabilizing transformed. The blue dotted line indicates 95th percentile.

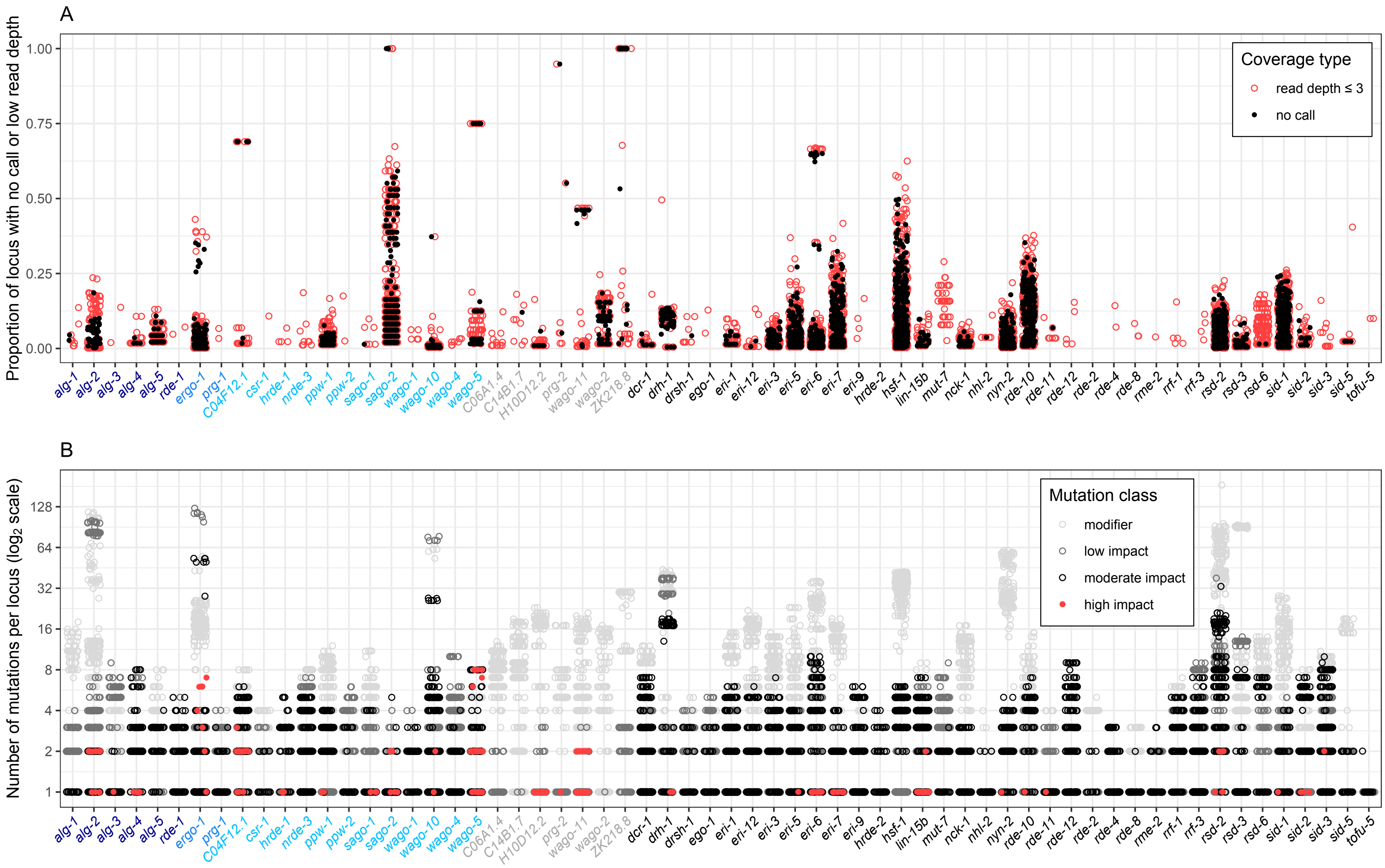

Figure S8. Population-level sequence variation for 62 RNAi genes. (A) mapping coverage and (B) mutational variation is shown for the 403 strain isotypes from CeNDR. Gene names are color coded by classification: AGO Argonaute (dark blue), PIWI Argonaute (blue), WAGO Argonaute (light blue), Argonaute pseudogene (grey), and other RNAi factor (black). Each point corresponds to an individual strain; zeros (no observation of poor coverage or mutation relative to the reference) are not plotted.

Figure S9. Seven low-response strains chosen for the *ppw-1* complementation tests, highlighted on the species tree. Modified from the species tree built from the 20200815 CeNDR release, available at https://elegansvariation.org/data/release/20200815. Strain JU1522, used in the study, is represented by isotype JU1581. *As this figure is very large, please see the file uploaded separately from this document.*

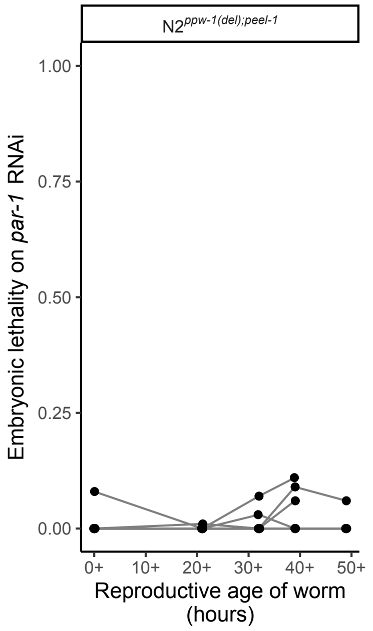

Figure S10. Embryonic lethality following RNAi against *par-1* in the *ppw-1;peel-2* mutant.

Table S1. Statistical results for RNAi in individual worms over lifespan (low-response strains).

Full model

	Df	Sum Sq	Mean Sq	F value	p-value
Strain	2	1.2934	0.64669	26.1440	<0.0001
Age	1	0.9190	0.91898	37.1523	<0.0001
Strain*Age	2	0.7757	0.38783	15.6793	<0.0001
Worm(Strain)	23	3.1750	0.13804	5.5807	<0.0001
Residuals	77	1.9046	0.02474		

Reduced model: CB4856

	Df	Sum Sq	Mean Sq	F value	p-value
Age	1	1.26666	1.26666	24.0905	<0.0001
Worm	9	0.36647	0.04072	0.7744	NS
Residuals	23	1.20932	0.05258		

Reduced model: QX1211

	Df	Sum Sq	Mean Sq	F value	p-value
Age	1	0.42788	0.42788	10.133	<0.0001
Worm	5	2.79942	0.55988	13.259	<0.0001
Residuals	16	0.67564	0.04223		

Table S2. Statistical estimates for changes in smFISH transcript abundance. The interaction term represents the changes in slope (Figure 2) between treated and untreated samples.

		Variance explained (ω^2)	p-value (ANCOVA model comparison)
N2	Treatment	0.586	<0.001
	Embryo stage	0.248	<0.001
	Interaction	0.059	<0.001
CB4856	Treatment	0.010	0.043
	Embryo stage	0.581	<0.001
	Interaction	0.017	0.012
QX1211	Treatment	0.014	0.037
	Embryo stage	0.208	<0.001
	Interaction	NA	NS

Table S3. Statistical results for the ddPCR analysis. The model used for each gene is described in the Methods.

ppw-1

	Df	Deviance	Resid. Df	Resid. Dev
NULL			158	37942
Strain	9	24433.1	149	13509
DevStage	1	910.3	148	12599
Strain:DevStage	9	4271.9	139	8327
Strain:DevStage:BiolRepl	20	2327.6	119	5999

sago-2

	Df	Deviance	Resid. DF	Resid. Dev
NULL			158	4722.0
RunDate	1	29.38	157	4692.6
Strain	9	2932.73	148	1759.9
DevStage	1	5.87	147	1754.0
Strain:DevStage	9	825.20	138	928.8
Strain:DevStage:BiolRepl	20	436.26	118	492.6

Table S4. Candidate genes for weak germline RNAi in the seven strains tested. Only genes with relevant genotypes in at least one strain are displayed, out of 62 RNAi genes queried. “High impact” indicates disruptive mutations like frameshifts or stop-gains. “Poor mapping coverage” indicates a read depth of 3 or less. “Functional diverged allele” indicates at least 1% nucleotide divergence from the reference genome, including at least five moderate mutations, such as amino acid substitutions, and no high impact mutations. Within this dataset, only *ergo-1* in strain ECA369 is located within a designated interval of hyper-diversity (Lee et al., 2021).

Class	Gene	One or more high impact mutation	Poor mapping coverage at 10% or more of locus	Functional diverged allele
Argonaute (AGO)	*alg-4*			QX1211
Argonaute (PIWI)	*ergo-1*	ECA369	ECA369	
Argonaute (WAGO)	*hrde-1*			QX1211
	nrde-3			QX1211
	ppw-1	CB4856		
	sago-2	DL238	DL238, ECA369, JU1522, KR314, QX1211	
	wago-10			QX1211
Argonaute (pseudogene)	*wago-11*	CB4852, KR314, QX1211	CB4856	
	wago-2		QX1211	
	ZK218.8		DL238	
Other RNAi factor	*dcr-1*			QX1211
	drh-1			QX1211
	eri-1			QX1211
	eri-5		JU1522	
	eri-6			QX1211
	eri-7	CB4856, DL238, ECA369	DL238, JU1522, KR314	
	eri-9			QX1211
	lin-15b	QX1211		ECA369
	nyn-2		DL238	QX1211
	rde-10		DL238, ECA369, JU1522, KR314, QX1211	
	rde-12			QX1211
	rsd-2		JU1522, QX1211	CB4856, DL238, ECA369, QX1211
	sid-1		DL238, ECA369, JU1522, QX1211	
	sid-2			QX1211
	sid-3			QX1211
	hsf-1		DL238, ECA369, JU1522, KR314	ECA369, KR314

Table S5. Estimates of nucleotide diversity

		RNAi genes		Other genes		Mann Whitney U	
Include pseud.	Include hyperdiv.	Median π per site	No. genes	Median π per site	No. genes	W	P-value
Yes	Yes	6.06E-04	62	7.00E-04	22,048	580,452	0.0401
No	Yes	5.01E-04	55	6.95E-04	19,931	432,488	0.0068
Yes	No	6.06E-04	52	6.96E-04	16,248	344,914	0.0221
No	No	5.00E-04	46	6.92E-04	15,183	266,041	0.0052

Table S6. Haplotype diversity metrics for RNAi genes. Genes located in an interval of hyper-diversity (Lee et al., 2021) in at least one strain are indicated by an asterisk.

Class	Gene	Haplotype diversity
Argonaute (AGO)	_alg-1_	0.864
	alg-2*	0.836
	alg-3	0.692
	alg-4	0.600
	alg-5	0.431
	rde-1	0.507
Argonaute (PIWI)	_ergo-1*_	0.954
	prg-1	0.410
Argonaute (WAGO)	_C04F12.1_	0.410
	csr-1	0.462
	hrde-1	0.381
	nrde-3	0.752
	ppw-1	0.761
	ppw-2	0.477
	sago-1	0.585
	sago-2	0.991
	wago-1	0.302
	wago-10*	0.868
	wago-4	0.912
	wago-5	0.736
Argonaute (pseudo)	_C06A1.4_	0.853
	_C14B1.7_	0.854
	_H10D12.2_	0.547
	prg-2	0.432
	wago-11	0.892
	wago-2	0.614
	_ZK218.8*_	0.494
Other RNAi factor	_dcr-1_	0.926
	drh-1*	0.557
	drsh-1	0.683
	ego-1	0.411

Class	Gene	Haplotype diversity
Other RNAi factor	_eri-1_	0.824
	eri-12	0.730
	eri-3	0.863
	eri-5	0.992
	eri-6	0.917
	eri-7	0.928
	eri-9	0.718
	hrde-2	0.434
	hsf-1	0.985
	lin-15b	0.865
	mut-7	0.810
	nck-1	0.705
	nhl-2	0.614
	nyn-2	0.955
	rde-10	0.949
	rde-11	0.601
	rde-12	0.701
	rde-2	0.299
	rde-4	0.488
	rde-8	0.429
	rme-2	0.316
	rrf-1	0.428
	rrf-3	0.892
	rsd-2*	0.958
	rsd-3*	0.844
	rsd-6	0.891
	sid-1	0.947
	sid-2	0.776
	sid-3	0.892
	sid-5	0.337
	tofu-5	0.169

Table S7. Strains used in this study.

Ref in text	Name	Genotype	Provenance
CB4852	CB4852	wild-type	Gift from Matthew Rockman
CB4856	CB4856	wild-type	Gift from Matthew Rockman
DL238	DL238	wild-type	Gift from Matthew Rockman
ECA369	ECA369	wild-type	Purchased from CeNDR
ECA701	ECA701	wild-type	Purchased from CeNDR
KR314	KR314	wild-type	Gift from Matthew Rockman
EG4348	EG4348	wild-type	Gift from Matthew Rockman
JU561	JU561	wild-type	Purchased from CeNDR
JU1088	JU1088	wild-type	Gift from Matthew Rockman
JU1522	JU1522	wild-type	Gift from Matthew Rockman
N2	N2	wild-type	Gift from Matthew Rockman
$N2^{peel\text{-}1}$	PTM377	*peel-1*(*kah126*) I	Gift from Patrick McGrath
$N2^{ppw\text{-}1}$	QF201	*ppw-1*(*pk1425*) I	Derived from backcrossing strain NL3511 5x to N2
$N2^{ppw\text{-}1;peel\text{-}1}$	QF204	*ppw-1*(*pk1425*) *peel-1*(*kah126*) I	Derived from PTM377 and QF201
$N2^{ppw\text{-}1\ CB4856}$	NL2550	*ppw-1*(*pk2505*) I	Purchased from CGC
NL3511	NL3511	*ppw-1*(*pk1425*) I	Purchased from CGC
QF14	QF14	*zuIs178*; *stIs10024 [RW10029>CB4856]*	Derived from backcrossing strain RW10029 18x to CB4856
QF15	QF15	*zuIs178; stIs10024 [RW10029>JU1522]*	Derived from backcrossing strain RW10029 16x to JU1522
QF16	QF16	*zuIs178*; *stIs10024 [RW10029>ECA369]*	Derived from backcrossing strain RW10029 17x to ECA369
QF90	QF90	*zuIs178*; *stIs10024 [RW10029>QX1211]*	Derived from backcrossing strain RW10029 10x to QX1211
QX1211	QX1211	wild-type	Gift from Matthew Rockman
RW10029	RW10029	*zuIs178*; *stIs10024*	Purchased from CGC
XZ1516	XZ1516	wild-type	Purchased from CeNDR

File S1. Statistical details of smFISH in early stage embryos

In early stage embryos (up to four cells), N2 showed a significant reduction in *par-1* transcript abundance after *par-1* RNAi ($t=-16.34$, $df=32$, $p<0.001$), while CB4856 and QX1211 did not ($t=0.41$, $df=56.31$, $p=0.684$ and $W=2145.5$, $p=0.9934$, respectively) (Figure 2D). However, for CB4856, the variance in abundance was slightly higher for treated embryos than for control embryos ($F=1.94$, $df=32,40$, $p=0.048$), which may represent the onset of an RNAi response. In QX1211, the distribution of transcript counts was multimodal for RNAi-treated embryos (but not untreated embryos) in both the *par-1* and *par-4* experiments (Excess mass test, $p<0.01$ for both), likely reflecting the subset of individuals with sensitive, N2-like RNAi responses. In the *par-4* experiment for QX1211, these low counts contributed to a slight reduction in median abundance relative to the untreated embryos ($W=249$, $p=0.0123$) (Figure 2D).

File S2. Details of genetic incompatibilities

C. elegans carries two known genetic incompatibilities: the paternally-delivered toxin *peel-1*, which is rescued by the zygotically-expressed antidote *zeel-1* (Seidel et al., 2008, 2011), and the maternally-delivered toxin *sup-35*, which is rescued by the zygotically-expressed antidote *pha-1* (Ben-David et al., 2017). In both instances, embryos that cytologically inherit the toxin but do not inherit the genotype to express the antidote will die. N2 carries both toxin-antidote complexes but several of our other wild strains do not. Since our goal was to use embryonic lethality to measure the RNAi response, lethality arising from these genetic incompatibilities had the potential to confound our results.

To control for the *zeel-1;peel-1* incompatibility, we generated a strain derived from N2 with both the *ppw-1* deletion and an allele of *peel-1* (*kah126*) that disables the toxin by a frameshift insertion in the second exon ($N2^{ppw\text{-}1(del);peel\text{-}1}$). This allele eliminates embryonic lethality that would otherwise arise in the F2 generation, from F1 heterozygotes derived from incompatible strains. The presence of this *peel-1* allele had no effect on our measured RNAi response in either sensitive or resistant backgrounds (Figure 3A, Figure S10), so we used it in all comparisons requiring the N2 background.

We did not control for the *sup-35;pha-1* incompatibility. We anticipated toxin-associated embryonic lethality to occur from crosses initiated between N2 and two wild strains without the active *sup-35;pha-1* complex: DL238 and QX1211. For DL238, we observed only very weak penetrance of this effect (Figure 3C). For QX1211, we observed clear toxin-associated lethality, as embryos derived from the N2 × QX1211 cross showed lethality on the control condition (Figure 3D). However, we were still able to infer a distinct *ppw-1*-associated response in this assay, as lethality from the $QX1211/N2^{peel\text{-}1}$ genotype matched that of the control and lethality from $QX1211/N2^{ppw\text{-}1(del);peel\text{-}1}$ was significantly higher (Figure 3D).
