# Supplementary material for: Diversification of small RNA pathways underlies germline RNA interference incompetence in wild Caenorhabditis elegans strains
Source: Genetics. 2023 Oct 22;226(1):iyad191. doi: 10.1093/genetics/iyad191 (PMC10763538; doi:10.1093/genetics/iyad191)
Supplement: iyad191_Supplementary_Data [file iyad191_supplementary_data.zip › File_S3_GENETICS-2023-306148.pdf]

CLUSTAL W (1.81) multiple sequence alignment

```
QX1211_ppw-1        -GTCTCTCGAACCATGGAAAAACAACTAGAAGCTATGTCCGTCTCGGACAAACCTGCTGC
CB4856_ppw-1        -GTCTCTCGAACCATGGAAAAACAACTAGAAGCTATGTCCGTCTCGGACAGACCTGCTGC
N2_ppw-1            AGTCTCTCGAACCATGGAAAAACAACTAGAAGCTATGTTCGTCTCGGACAGACCTGCTGC
N2_sago-2           AGTCTCTCGAACTATGGAAAAACAACTAAAAGCTATGTCCGTCTCGGACAAACCTGCTGC
CB4856_sago-2       AGTCTCTCGAACTATGGAAAAACAACTAAAAGCTATGTCCGTCTCGGACAAACCTGCTGC
QX1211_sago-2       AGTCTCTCGAACTATGGAAAAACAACTAAAAGCTATGTCCGTCTCGGACAAACCTGCTGC
                     *********** *************** ********* *********** *********

QX1211_ppw-1        CCCAGCTGCCCAAAAGCTTGGTACCGCTCCGCTCGCTGCAAAAAAGACGAGAAATGTGGA
CB4856_ppw-1        CCCAGCTGCCCAAAAGCTTGGTACCGCTCCGCTCGCTGCAAAAAAGACGAGAAATGTGGA
N2_ppw-1            CCCAGCTGCCCAAAAGCTTGGTACCGCTCCGCTCGCTGCAAAAAAGACGAGAAATGTGGA
N2_sago-2           CCCAGCTGCCCAAAAGCTTGGTACCGCTCCGCTCGCTGCAAAAAAGACGAGAAATGAGGA
CB4856_sago-2       CCCAGCTGCCCAAAAGCTTGGTACCGCTCCGCTCGCTGCAAAAAAGACGAGAAATGAGGA
QX1211_sago-2       CCCAGCTGCCCAAAAGCTTGGTACCGCTCCGCTCGCTGCAAAAAAGACGAGAAATGAGGA
                    ******************************************************** ***

QX1211_ppw-1        GAGGGGAACCAAGGTCAATATCGATACCAAC---ATTCGCAAATTGAATAAGTTTTTATT
CB4856_ppw-1        GAGGGGAACCAAGGTCAATATCGATACCAACATTATTCGCAAATTGAATAAGTTTTTATT
N2_ppw-1            GAGGGGAACCAAGGTCAATATCGATACCAAC---ATTCGCAAATTGAATAAGTTTTTATT
N2_sago-2           GTGGGGAACCAAGGTCAATATCGATACCAAC---ATTCGCAAATTGAGTAAGTTTTTATT
CB4856_sago-2       GTGGGGAACCAAGGTCAATATCGATACCAAC---ATTCGCAAATTGAGTAAGTTTTTATT
QX1211_sago-2       GTGGGGAACCAAGGTCAATATCGATACCAAC---ATTCGCAAATTGAGTAAGTTTTTATT
                    * ****************************   ************* *************

QX1211_ppw-1        TTACAATTACCAGTCGTTCGAGCTTACATATCAATATTTTTCAGCGATCAAACCGAATCA
CB4856_ppw-1        TTACAATTACCAGTCGTTCGAGCTTACATATCAATATTTTTCAGCGATCAAACCGAATCA
N2_ppw-1            TTACAATTACGAGTCGTTCGAGCTCACATATCAATATTTTTCAGCGATCAAACCGAATCA
N2_sago-2           TTACAATTACCAGTCGTTCGAGCTTATATATCAATATTTTTTAGCGATCAAACCGAATCA
CB4856_sago-2       TTACAATTACCAGTCGTTCGAGCTTATATATCAATATTTTTTAGCGATCAAACCGAATCA
QX1211_sago-2       TTACAATTACCAGTCGTTCGAGCTTATATATCAATATTTTTTAGCGATCAAACCGAATCA
                    ********** ************* * ************** ******************

QX1211_ppw-1        GCCAATTTACAAGTACGCTGTGCAAGTGAACTACGTCTTCCGGAAACCTGATGGAACTGA
CB4856_ppw-1        GCCAATTTACAAGTACGCTGTGCAAGTGAACTACGTCTTCCGGAAACCTGATGGAACTGA
N2_ppw-1            GCCAATTTACAAGTACGCTGTGCAAGTGAACTACGTCTTCCGGAAACCTGATGGAACTGA
N2_sago-2           GCCAATTTACAAGTACGCTGTGCAAGTGAACTACGTCTTCCGGAAACCTGATGGAACTGA
CB4856_sago-2       GCCAATTTACAAGTACGCTGTGCAAGTGAACTACGTCTTCCGGAAACCTGATGGAACTGA
QX1211_sago-2       GCCAATTTACAAGTACGCTGTGCAAGTGAACTACGTCTTCCGGAAACCTGATGGAACTGA
                    ************************************************************

QX1211_ppw-1        GGCGACAATCGAAATGTCCAAATCAGCCAAAAAGGGAACGGAGCACGACAACGACAAAAC
CB4856_ppw-1        GGCGACAATCGAAATGTCCAAATCAGCCAAAAAGGGAACGGAGCACGACAACGACAAAAC
N2_ppw-1            GGCGACAATCGAAATGTCCAAATCAGCCAAAAAGGGAACGGAGCACGACAACGACAAAAC
N2_sago-2           GGCGACAATCGAAATGTCCAAATCAGCCAAAAAGGGAACGGAGCACGACAACGACAAAAC
CB4856_sago-2       GGCGACAATCGAAATGTCCAAATCAGCCAAAAAGGGAACGGAGCACGACAACGACAAAAC
QX1211_sago-2       GGCGACAATCGAAATGTCCAAATCAGCCAAAAAGGGAACGGAGCACGACAACGACAAAAC
                    ************************************************************

QX1211_ppw-1        ACGCTGCCAGAAGGTTTACAATGAGGCGATCAAGCGTTACGATGAGCTGAAAACCGGAG-
CB4856_ppw-1        ACGCTGCCAGAAGGTTTACAATGAGGCGATCAAGCGTTACGATGAGCTGAAAACCGGAGG
N2_ppw-1            ACGCTGCCAGAAGGTTTACAATGAGGCGATCAAGCGTTACGATGAGCTGAAAACCGGAGG
N2_sago-2           ACGCTGCCAGAACGTTTACAATGAGGCGATCAAGCGTTACGATGAGCTGAAAACCGGAG-
CB4856_sago-2       ACGCTGCCAGAATGTTTACAATGAGGCGATCAAGCGTTACGATGAGCTGAAAACCGGAG-
QX1211_sago-2       ACGCTGCCAGAACGTTTACAATGAGGCGATCAAGCGTTACGATGAGCTGAAAACCGGAG-
                    ************ ********************************************** 

QX1211_ppw-1        ------------------------------------------------------------
CB4856_ppw-1        ACCGTTTTTCTACGATCGTCAAGCCTCTTTGTACACTCTGACCAAATTGAAGAATGAGGT
N2_ppw-1            ACCGTTTTTCTACGATCGTCAAGCCTCTTTGTACACTCTGACCAAATTGAAGAATGAGGT
```

```
N2_sago-2           ------------------------------------------------------------
CB4856_sago-2       ------------------------------------------------------------
QX1211_sago-2       ------------------------------------------------------------


QX1211_ppw-1        -------------------------------------GACCGTTTTTCTACGATCGTCAA
CB4856_ppw-1        GAATTCGATCAAGCGTTACGATGAGCTGAAAACCGGAGACCGTTTTTCTACGATCGTCAA
N2_ppw-1            GAATTCGATCAAGCGTTACGATGAGCTGAAAACCGGAGACCGTTTTTCTACGATCGTCAA
N2_sago-2           -------------------------------------GACCGTTTTTCTACGATCGTCAA
CB4856_sago-2       -------------------------------------GACCGTTTTTCTACGATCGTCAA
QX1211_sago-2       -------------------------------------GACCGTTTTTCTACGATCGTCAA
                                                         ***********************

QX1211_ppw-1        GCCTCTTTGTACACTCTGACCAAATTGAAGAATGAGGTGAATTCGATTTTTTGCTTAAAG
CB4856_ppw-1        GCCTCTTTGTACACTCTGACCAAATTGAAGAATGAGGTGAATTCGATTTTTTGCTTAAAG
N2_ppw-1            GCCTCTTTGTACACTCTGACCAAATTGAAGAATGAGGTGAATTCGATTTTTTGCTTAAAG
N2_sago-2           GCCTCTTTGTACACTCTGACCAAATTGAAGAATGAGGTGAATTCGATTTTTTGCTTAAAG
CB4856_sago-2       GCCTCTTTGTACACTCTGACCAAATTGAAGAATGAGGTGAATTCGATTTTTTGCTTAAAG
QX1211_sago-2       GCCTCTTTGTACACTCTGACCAAATTGAAGAATGAGGTGAATTCGATTTTTTGCTTAAAG
                    ************************************************************

QX1211_ppw-1        TTTATAATTTTGCGTTTCAGAGCATCTCTTTCGTTGTTACTGACAAGATTTGCAAGCGAC
CB4856_ppw-1        TTTAATATTTTGCGTTTCAGAGCATCTCTTTCGTTGTTACTGACAAGATTTGCAAGCGAC
N2_ppw-1            TTTAATATTTTGCGTTTCAGAGCATCTCTTTCGTTGTTACTGACAAGATTTGCAAGCGAC
N2_sago-2           TTTAATATTTTGCGTTTCAGAGCATCTCTTTCGTTGTTACTGACAAGATTTGCAAGCGAC
CB4856_sago-2       TTTAATATTTTGCGTTTCAGAGCATCTCTTTCGTTGTTACTGACAAGATTTGCAAGCGAC
QX1211_sago-2       TTTAATATTTTGCGTTTCAGAGCATCTCTTTCGTTGTTACTGACAAGATTTGCAAGCGAC
                    ****  ******************************************************

QX1211_ppw-1        AAAACTTCAAAGAGGCACAGTTTGTTCTCAAAAAGGTTGATCAATCGTTCCAGTCAACAT
CB4856_ppw-1        AAAACTTCAAAGAGGCACAGTTTGTTCTCAAAAAGGTTGATCAATCGTTCCAGTCAACAT
N2_ppw-1            AAAACTTCAAAGAGGCACAGTTTGTTCTCAAAAAGGTTGATCAATCGTTCCAGTCAACAT
N2_sago-2           AAAACTTCAAAGAGGCACAGTTTGTTCTCAAAAAGGTGGATCAATCGTTCCAGTCAACAT
CB4856_sago-2       AAAACTTCAAAGAGGCACAGTTTGTTCTCAAAAAGGTGGATCAATCGTTCCAGTCAACAT
QX1211_sago-2       AAAACTTCAAAGAGGCACAGTTTGTTCTCAAAAAGGTTGATCAATCGTTCCAGTCAACAT
                    ************************************* **********************

QX1211_ppw-1        CGAATGACGTCATCAGGACAACCAACTCGTGCCCAGCCAATGCCGACAAAACTTTGCTTG
CB4856_ppw-1        CGAATGACGTCATCAGGACAACCAACTCGTGCCCAGCCAATGCCGACAAAACTTTGCTTG
N2_ppw-1            CGAATGACGTCATCAGGACAACCAACTCGTGCCCAGCCAATGCCGACAAAACTTTGCTTG
N2_sago-2           CGAATGACGTCATCAAGACAACCAACTCGTGCCCAGCCAATGCCGACAAAACTTTGCTTG
CB4856_sago-2       CGAATGACGTCATCAAGACAACCAACTCGTGCCCAGCCAATGCCGACAAAACTTTGCTTG
QX1211_sago-2       CGAATGACGTCATCAGGACAACCAACTCGTGCCCAGCCAATGCCGACAAAACTTTGCTTG
                    *************** ********************************************

QX1211_ppw-1        AGGCAATGAACATCATTGTCTCGGGACCAGCGTTCGAAAAGTATGCCGCTTTCAGAGATA
CB4856_ppw-1        AGGCAATGAACATCATTGTCTCGGGACCAGCGTTCGAAAAGTATGCCGCTTTCAGAGATA
N2_ppw-1            AGGCAATGAACATCATTGTCTCGGGACCAGCGTTCGAAAAGTATGCCGCTTTCAGAGATA
N2_sago-2           AGGCAATGAACATCATTGTCTCGGGACCAGCGTTCGAAAAGTATGCCGCTTTCAGAGATA
CB4856_sago-2       AGGCAATGAACATCATTGTCTCGGGACCAGCGTTCGAAAAGTATGCCGCTTTCAGAGATA
QX1211_sago-2       AGGCAATGAACATCATTGTCTCGGGACCAGCGTTCGAAAAGTATGCCGCTTTCAGAGATA
                    ************************************************************

QX1211_ppw-1        ATCAAGCTATTTCATCTCGTTTTCAGCAAAAATGTTATCACCGTTGGAGCATGCGTTCAT
CB4856_ppw-1        ATCAAGCTATTTCATCTCGTTTTCAGCAAAAATGTTATCACCGTTGGAGCATGCGTTCAT
N2_ppw-1            ATCAAGCTATTTCATCTCGTTTTCAGCAAAAATGTTATCACCGTTGGAGCATGCGTTCAT
N2_sago-2           ATCAAGCTATTTCATCTCGTTTTCAGCAAAAATGTTATCACCGTTGGAGCATGCGTTCAT
CB4856_sago-2       ATCAAGCTATTTCATCTCGTTTTCAGCAAAAATGTTATCACCGTTGGAGCATGCGTTCAT
QX1211_sago-2       ATCAAGCTATTTCATCTCGTTTTCAGCAAAAATGTTATCACCGTTGGAGCATGCGTTCAT
                    ************************************************************
```

```
QX1211_ppw-1      TACCTCATCGACCCAACTGTAGTCGAGTAAGAAAC--TAAGAAATTATCGTATTAGTATT
CB4856_ppw-1      TACCTCATCGACCCAGCTGTAGTCGAGTAAGAAAC--TAAGAAATTATCGTATTAGTATT
N2_ppw-1          TACCTCATCGACCCAACTGTAGTCGAGTAAGAAAC--TAAGAAATTATCGTATTAGTATT
N2_sago-2         TACCTCATCGACCCAACTGGAGTCGAGTAAGAAACTTTAAGAAATTATCATATTAGTATT
CB4856_sago-2     TACCTCATCGACCCAACTGGAGTCGAGTAAGAAACTTTAAGAAATTATCATATTAGTATT
QX1211_sago-2     TACCTCATCGACCCAACTGGAGTCGAGTAAGAAAC--TAAGAAATTATCGTATTAGTATT
                  *************** *** ***************  ************ **********

QX1211_ppw-1      TAATTGTTTTTTCCAGCGTCGCGTACAAGGAATACGCTGAAGGACAACTCTACTCAGGAG
CB4856_ppw-1      TAATTGTTTTTTCCAGCGTCGCGTACAAGGAATACGCTGAAGGACAACTCTACTCAGGAG
N2_ppw-1          TAATTGTTTTTTCCAGCGTCGCGTACAAGGAATACGCTGAAGGACAACTCTACTCAGGAG
N2_sago-2         TAATTGTTTTTTCCAGCGTCGCGTACAAGGAATACCCTGAAGGACAACTCTACTCAGGAG
CB4856_sago-2     TAATTGTTTTTTCCAGCGTCGCGTACAAGGAATACCCTGAAGGACAACTCTACTCAGGAG
QX1211_sago-2     TAATTGTTTTTTCCAGCGTCGCGTACAAGGAATACGCTGAAGGACAACTCTACTCAGGAG
                  ********************************** *************************

QX1211_ppw-1      TTGGTGCTTCAAAGTCGGTGAAGACATTGGAAGGAACGGACAAGAAAGTTCCATCACTCT
CB4856_ppw-1      TTGGTGCTTCAAAGTCGGTGAAGACATTGGAAGGAACGGACAAGAAAGTTCCATCACTCT
N2_ppw-1          TTGGTGCTTCAAAGTCGGTGAAGACATTGGAAGGAACGGACAAGAAAGTTCCATCACTCT
N2_sago-2         TTGGTGTTTCAAAGTCGGTGAAGACATTGGAAGGAACGGACAAGAAAGTTCCATCACTCT
CB4856_sago-2     TTGGTGTTTCAAAGTCGGTGAAGACATTGGAAGGAACGGACAAGAAAGTTCCATCACTCT
QX1211_sago-2     TTGGTGCTTCAAAGTCGGTGAAGACATTGGAAGGAACGGACAAGAAAGTTCCATCACTCT
                  ****** *****************************************************

QX1211_ppw-1      TCATGACAACTGAAAGTTAGTTTTTCAGAAAATCTAGA---TTTTCAAAATGTTATAAAA
CB4856_ppw-1      TCATGA-AACTGAAAGTTAGTTTTTCAGAAAATCTAGA---TTTTCAAAATGTTATAAAA
N2_ppw-1          TCATGACAACTGAAAGTTAGTTTTTCAGAAAATCTAGA---TTTTCAAAATGTTATAAAA
N2_sago-2         TCATGACAACTGAAAGTTAGTTTTTCAGAAAATCTAGATTTTTTTCAAAATGTTATAAAA
CB4856_sago-2     TCATGACAACTGAAAGTTAGTTTTTCAGAAAATCTAGATTTTTTTCAAAATGTTATAAAA
QX1211_sago-2     TCATGACAACTGAAAGTTAGTTTTTCAGAAAATCTAGA---TTTTCAAAATGTTATAAAA
                  ****** *******************************   *******************

QX1211_ppw-1      TTGTTAATTTTCAGTGAAGACAACATTGTTCCATCCGGATTATGCCCCACTTGTGGAACT
CB4856_ppw-1      TTGTTAATTTTCAGTGAAGACAACATTGTTCCATCCGGATTATGCCCCACTTGTGGAACT
N2_ppw-1          TTGTTAATTTTCAGTGAAGACAACATTGTTCCATCCGGATTATGCCCCACTTGTGGAACT
N2_sago-2         TTGTTAATTTTCAGTGAAGACAACATTGTTCCATCCGGATTATGCCCCACTTGTGGAACT
CB4856_sago-2     TTGTTAATTTTCAGTGAAGACAACATTGTTCCATCCGGATTATGCCCCACTTGTGGAACT
QX1211_sago-2     TTGTTAATTTTCAGTGAAGACAACATTGTTCCATCCGGATTATGCCCCACTTGTGGAACT
                  ************************************************************

QX1211_ppw-1      TTTGCAAACGTTCAGAGGTTTCAGCACAACTCTCAAAGCGAATTCTCCAGCCGCGCAGAG
CB4856_ppw-1      TTTGCAAACGTTCAGAGGTTTCAGCACAACTCTCAAAGCGAATTCTCCAGCCGCGCAGAG
N2_ppw-1          TTTGCAAACGTTCAGAGGTTTCAGCACAACTCTCAAAGCGAATTCTCCAGCCGCGCAGAG
N2_sago-2         TTTGCAAACGTTCAGAGGTTTCAGCACAACTCTCAAAGCGAATTCTCCAGCCGCGCAGAG
CB4856_sago-2     TTTGCAAACGTTCAGAGGTTTCAGCACAACTCTCAAAGCGAATTCTCCAGCCGCGCAGAG
QX1211_sago-2     TTTGCAAACGTTCAGAGGTTTCAGCACAACTCTCAAAGCGAATTCTCCAGCCGCGCAGAG
                  ************************************************************

QX1211_ppw-1      AATTGAGAAAGCCTTTGTTGGACTGGATGTTGTCTTGAATTACGGTGTGCACAAGGGTCT
CB4856_ppw-1      AATTGAGAAAGCCTTTGTTGGACTGGATGTTGTCTTGAATTACGGTGTGCACAAGGGTCT
N2_ppw-1          AATTGAGAAAGCCTTTGTTGGACTGGATGTTGTCTTGAATTACGGTGTGCACAAGGGTCT
N2_sago-2         AATTGAGAAAGCCTTTGTTGGACTGGATGTTGTCTTGAATTACGGTGTGCACAAGGGTCT
CB4856_sago-2     AATTGAGAAAGCCTTTGTTGGACTGGATGTTGTCTTGAATTACGGTGTGCACAAGGGTCT
QX1211_sago-2     AATTGAGAAAGCCTTTGTTGGACTGGATGTTGTCTTGAATTACGGTGTGCACAAGGGTCT
                  ************************************************************

QX1211_ppw-1      CGGAGAGGATGGTGTCGTTATGAAGATCCGTCGATTCCACACGTCAGCTAAGGAGACATG
CB4856_ppw-1      CGGAGAGGATGGTGTCGTTATGAAGATCCGTCGATTCCACACGTCAGCTAAGGAGACATG
N2_ppw-1          CGGAGAGGATGGTGTCGTTATGAAGATCCGTCGATTCCACACGTCAGCTAAGGAGACATG
N2_sago-2         CGGAGAGGATGGTGTCGTTATGAAGATCCGTCGATTCCACACGTCAGCTAAGGAGACATG
CB4856_sago-2     CGGAGAGGATGGTGTCGTTATGAAGATCCGTCGATTCCACACGTCAGCTAAGGAGACATG
```

```
QX1211_sago-2      CGGAGAGGATGGTGTCGTTATGAAGATCCGTCGATTCCACACGTCAGCTAAGGAGACATG
                   ************************************************************

QX1211_ppw-1       TTTTGAAGTTGAGAAGTCAACTCGTGAATTCACGAACGTCTTTGACTACTTCAAAAAGAA
CB4856_ppw-1       TTTTGAAGTTGAGAAGTCAACTCGTGAATTCACGAACGTCTTTGACTACTTCAAAAAGAA
N2_ppw-1           TTTTGAAGTTGAGAAGTCAACTCGTGAATTCACGAACGTCTTTGACTACTTCAAAAAGAA
N2_sago-2          TTTTGAAGTTGAGAAGTCAACTCGTGAATTCACGAACGTCTTTGACTACTTCAAAAAGAA
CB4856_sago-2      TTTTGAAGTTGAGAAGTCAACTCGTGAATTCACGAACGTCTTTGACTACTTCAAAAAGAA
QX1211_sago-2      TTTTGAAGTTGAGAAGTCAACTCGTGAATTCACGAACGTCTTTGACTACTTCAAAAAGAA
                   ************************************************************

QX1211_ppw-1       GTATGGAATCACTTTGAAGTATCCCGATTTATTCACCATTGAAGCGAAAGGGAAACAAGG
CB4856_ppw-1       GTATGGAATCACTTTGAAGTATCCCGATTTATTCACCATTGAAGCGAAAGGGAAACAAGG
N2_ppw-1           GTATGGAATCACTTTGAAGTATCCCGATTTATTCACCATTGAAGCGAAAGGGAAACAAGG
N2_sago-2          GTATGGAATCACTTTGAAGTATCCCGATTTATTCACCATTGAAGCGAAAGGGAAACAAGG
CB4856_sago-2      GTATGGAATCACTTTGAAGTATCCCGATTTATTCACCATTGAAGCGAAAGGGAAACAAGG
QX1211_sago-2      GTATGGAATCACTTTGAAGTATCCCGATTTATTCACCATTGAAGCGAAAGGGAAACAAGG
                   ************************************************************

QX1211_ppw-1       AAAAATTCATTTCCCTGCGGAAGTTCTCCTTCTCTGTCCGAACCAGACGGTCACGAATGA
CB4856_ppw-1       AAAAATTCATTTCCCTGCGGAAGTTCTCCTTCTCTGTCCGAACCAGACGGTCACGAATGA
N2_ppw-1           AAAAATTCATTTCCCTGCGGAAGTTCTCCTTCTCTGTCCGAACCAGACGGTCACGAATGA
N2_sago-2          AAAAATTCATTTCCCTGCGGAAGTTCTCCTTCTCTGTCCGAACCAGACGGTCACGAATGA
CB4856_sago-2      AAAAATTCATTTCCCTGCGGAAGTTCTCCTTCTCTGTCCGAACCAGACGGTCACGAATGA
QX1211_sago-2      AAAAATTCATTTCCCTGCGGAAGTTCTCCTTCTCTGTCCGAACCAGACGGTCACGAATGA
                   ************************************************************

QX1211_ppw-1       TCAAATGATCAACAATGAGCAGGCGGACATGATTAAGGTAATTTTATATTTTAAAATCTG
CB4856_ppw-1       TCAAATGATCAACAATGAGCAGGCGGACATGATTAAGGTAATTTTATATTTTAAAATCTG
N2_ppw-1           TCAAATGATCAACAATGAGCAGGCGGACATGATTAAGGTAATTTTATATTTTAAAATCTG
N2_sago-2          TCAAATGATCAACAATGAGCAGGCGGACATGATTAAGGTAATTTTATATTTTAAAATCTG
CB4856_sago-2      TCAAATGATCAACAATGAGCAGGCGGACATGATTAAGGTAATTTTATATTTTAAAATCTG
QX1211_sago-2      TCAAATGATCAACAATGAGCAGGCGGACATGATTAAGGTAATTTTATATTTTAAAATCTG
                   ************************************************************

QX1211_ppw-1       AATAATATCCCGAATCCTTTTTCAGATGTCAGCCGCACAACCACATATCAGAAAGACGAC
CB4856_ppw-1       AATAATATCCCGAATCCTTTTTCAGATGTCAGCCGCACAACCACATATCAGAAAGACGAC
N2_ppw-1           AATAATATCCCGAATCCTTTTTCAGATGTCAGCCGCACAACCACATATCAGAAAGACGAC
N2_sago-2          AATAATATCCCGAATCCTTTTTCAGATGTCAGCCGCACAACCACATATCAGAAAGACGAC
CB4856_sago-2      AATAATATCCCGAATCCTTTTTCAGATGTCAGCCGCACAACCACATATCAGAAAGACGAC
QX1211_sago-2      AATAATATCCCGAGTCCTTTTTCAGATGTCAGCCGCACAACCACATATCAGAAAGACGAC
                   ************* **********************************************

QX1211_ppw-1       AACTGATACTATCGTGAGAAACGTCGGATTGGCTTCCAACAATATCTATGGCTTCATCAA
CB4856_ppw-1       AACTGATACTATCGTGAGAAACGTCGGATTGGCTTCCAACAATATCTATGGCTTCATCAA
N2_ppw-1           AACTGATACTATCGTGAGAAACGTCGGATTGGCTTCCAACAATATCTATGGCTTCATCAA
N2_sago-2          AACTGATACTATCGTGAGAAACGTCGGATTGGCTTCCAACAATATCTATGGCTTCATCAA
CB4856_sago-2      AACTGATACTATCGTGAGAAACGTCGGATTGGCTTCCAACAATATCTATGGCTTCATCAA
QX1211_sago-2      AACTGATACTATCGTGAGAAACGTCGGATTGGCTTCCAACAATATCTATGGCTTCATCAA
                   ************************************************************

QX1211_ppw-1       AGTTGAAGACCCAGTCAACCTTGAAGGAATGGTTCTTCCAAAACCTAAGATTGCGTTCGC
CB4856_ppw-1       AGTTGAAGACCCAGTCAACCTTGAAGGAATGGTTCTTCCAAAACCTAAGATTGCGTTCGC
N2_ppw-1           AGTTGAAGACCCAGTCAACCTCGAAGGAATGGTTCTTCCAAAACCTAAGATTGCGTTCGC
N2_sago-2          AGTTGAAGACCCAGTCAACCTTGAAGGAATGGTTCTTCCAAAACCTAAGATTGCGTTTGC
CB4856_sago-2      AGTTGAAGACCCAGTCAACCTTGAAGGAATGGTTCTTCCAAAACCTAAGATTGCGTTTGC
QX1211_sago-2      AGTTGAAGACCCAGTCAACCTTGAAGGAATGGTTCTTCCAAAACCTAAGATTGCGTTCGC
                   ******************** ************************************ **

QX1211_ppw-1       CGGTAACCGACTCGCTGATTTGGCAAATCCGAAGTCTAGATTCCCCACCGACTTCAATCG
CB4856_ppw-1       CGGTAACCAACTCGCTGATTTGGCAAATCCGAAGTCTAGATTCCCCACCGACTTCAATCG
```

```
N2_ppw-1            CGGTAACCAACTCGCTGATTTGGCAAATCCGAAGTCTAGATTCCCCACCGACTTCAATCG
N2_sago-2           CGGTAACCGACTCGCTGATTTGGCAAATCCGAAGTCTAGATTCCCCACCGACTTCAATCG
CB4856_sago-2       CGGTAACCGACTCGCTGATTTGGCAAATCCGAAGTCTAGATTCCCCACCGACTTCAATCG
QX1211_sago-2       CGGTAACCGACTCGCTGATTTGGCAAATCCGAAGTCTAGATTCCCCACCGACTTCAATCG
                    ******** ***************************************************

QX1211_ppw-1        TGCTGGACAATATTACGATGCCAAGGAATTGACGAAATGGGAACTGGTCTTTGTTCAAAA
CB4856_ppw-1        TGCTGGACAATATTACGATGCCAAGGAATTGACGAAATGGGAACTGGTCTTTGTTCAAAA
N2_ppw-1            TGCTGGACAATATTACGATGCCAAGGAATTGACGAAATGGGAACTGGTCTTTGTTCAAAA
N2_sago-2           TGCTGGACAATATTACGATGCCAAGGAATTGACGAAATGGGAACTGGTCTTTGTTCAAAA
CB4856_sago-2       TGCTGGACAATATTACGATGCCAAGGAATTGACGAAATGGGAACTGGTCTTTGTTCAAAA
QX1211_sago-2       TGCTGGACAATATTACGATGCCAAGGAATTGACAAAATGGGAACTGGTCTTTGTTCAAAA
                    ********************************* **************************

QX1211_ppw-1        CGAAGAAGTCCAGTAAGTTTCAGTTTGTAATTTTTCATTTTATTGACTCAATTTCAGAGG
CB4856_ppw-1        CGAAGAAGTCCAGTAAGTTTCAGTTTGTAATTTTTCATTTTATTGACTCAATTTCAGAGG
N2_ppw-1            CGAAGAAGTCCAGTAAGTTTCAGTTTGTAATTTTTCATTTTATTGACTCAATTTCAGAGG
N2_sago-2           CGAAGAAGTCCAGTAAGTTTCAGTTTGTAATTTTTCATTTTATTGACTCAATTTCAGAGG
CB4856_sago-2       CGAAGAAGTCCAGTAAGTTTCAGTTTGTAATTTTTCATTTTATTGACTCAATTTCAGAGG
QX1211_sago-2       CGAAGAAGTCCAGTAAGTTTCAGTTTGTAATTTTTCATTTTATTGACTCAATTTCAGAGG
                    ************************************************************

QX1211_ppw-1        ACTTGCTAAGCAGCTCGCCGATGAAATGGTGAATAATGGTATGAAATGCAGCAACCCAAC
CB4856_ppw-1        ACTTGCTAAGCAGCTCGCCGATGAAATGGTGAATAATGGTATGAAATGCAGCAACCCAAC
N2_ppw-1            ACTTGCTAAGCAGCTCGCCGATGAAATGGTGAATAATGGTATGAAATGCAGCAACCCAAC
N2_sago-2           ACTTGCTAAGCAGCTCGCCGATGAAATGGTGAATAATGGTATGAAATGCAGCAACCCAAC
CB4856_sago-2       ACTTGCTAAGCAGCTCGCCGATGAAATGGTGAATAATGGTATGAAATGCAGCAACCCAAC
QX1211_sago-2       ACTTGCTAAGCAGCTCGCCGATGAAATGGTGAATAATGGTATGAAATGCAGCAACCCAAC
                    ************************************************************

QX1211_ppw-1        GATGAGTTTCATCATTAGAGGTGATTTGGAACCAATCTTCAAGAAAGCGAAGGCTGCTGG
CB4856_ppw-1        GATGAGTTTCATCATTAGAGGTGATTTGGAACCAATCTTCAAGAAAGCGAAGGCTGCTGG
N2_ppw-1            GATGAGTTTCATCATTAGAGGTGATTTGGAACCAATCTTCAAGAAAGCGAAGGCTGCTGG
N2_sago-2           GATGAGTTTCATCATTAGAGGTGATTTGGAACCAATCTTCAAGAAAGCGAAGGCTGCTGG
CB4856_sago-2       GATGAGTTTCATCATTAGAGGTGATTTGGAACCAATCTTCAAGAAAGCGAAGGCTGCTGG
QX1211_sago-2       GATGAGTTTCATCATTAGAGGTGATTTGGAACCAATCTTCAAGAAAGCGAAGGCTGCTGG
                    ************************************************************

QX1211_ppw-1        AACGCAACTTCTCTTCTTCGTTGTCAAATCTCGCTACAACTATCATCAGCAAATCAAGGC
CB4856_ppw-1        AACGCAACTTCTCTTCTTCGTTGTCAAATCTCGCTACAACTATCATCAGCAAATCAAGGC
N2_ppw-1            AACGCAACTTCTCTTCTTCGTTGTCAAATCTCGCTACAACTATCATCAGCAAATCAAGGC
N2_sago-2           AACGCAACTTCTCTTCTTCGTTGTCAAATCTCGTTACAACTATCATCAGCAAATCAAGGC
CB4856_sago-2       AACGCAACTTCTCTTCTTCGTTGTCAAATCTCGTTACAACTATCATCAGCAAATCAAGGC
QX1211_sago-2       AACGCAACTTCTCTTCTTCGTTGTCAAATCTCGCTACAACTATCATCAGCAAATCAAGGC
                    ********************************* **************************

QX1211_ppw-1        GTTGGAGCAAAAGTATGACGTGCTCACTCAGGAGATTCGCGCTGAAACCGCCGAGAAAGT
CB4856_ppw-1        GTTGGAGCAAAAGTATGACGTGCTCACTCAGGAGATTCGCGCTGAAACCGCCGAGAAAGT
N2_ppw-1            GTTGGAGCAAAAGTATGACGTGCTCACTCAGGAGATTCGCGCTGAAACCGCCGAGAAAGT
N2_sago-2           GTTGGAGCAAAAGTATGACGTGCTCACTCAGGAGATTCGCGCTGAAACCGCCGAGAAAGT
CB4856_sago-2       GTTGGAGCAAAAGTATGACGTGCTCACTCAGGAGATTCGCGCTGAAACCGCCGAGAAAGT
QX1211_sago-2       GTTGGAGCAAAAGTATGACGTGCTCACTCAGGAGATTCGCGCTGAAACCGCCGAGAAAGT
                    ************************************************************

QX1211_ppw-1        CTTCCGTCAACCACAAACTCGTCTTAACATTATCAATAAGACGAACATGAAACTCGGAGG
CB4856_ppw-1        CTTCCGTCAACCACAAACTCGTCTTAACATTATCAATAAGACGAACATGAAACTCGGAGG
N2_ppw-1            CTTCCGTCAACCACAAACTCGTCTTAACATTATCAATAAGACGAACATGAAACTCGGAGG
N2_sago-2           CTTCCGTCAACCACAAACTCGTCTTAACATTATCAATAAGACGAACATGAAACTCGGAGG
CB4856_sago-2       CTTCCGTCAACCACAAACTCGTCTTAACATTATCAATAAGACGAACATGAAACTCGGAGG
QX1211_sago-2       CTTCCGTCAACCACAAACTCGTCTTAACATTATCAATAAGACGAACATGAAACTCGGAGG
                    ************************************************************
```

```
QX1211_ppw-1      ACTGAACTATGCTATTGGGAGTGAAGCGTTCAACAAGCCAAATAGATTGATCGTTGGATT
CB4856_ppw-1      ACTGAACTATGCTATTGGGAGTGAAGCGTTCAACAAGCCAAATAGATTGATCGTTGGATT
N2_ppw-1          ACTGAACTATGCTATTGGGAGTGAAGCGTTCAACAAGCCAAATAGATTGATCGTTGGATT
N2_sago-2         ACTGAACTATGCTATTGGGAGTGAAGCGTTCAACAAGCCAAATAGATTGATCGTTGGATT
CB4856_sago-2     ACTGAACTATGCTATTGGGAGTGAAGCGTTCAACAAGCCAAATAGATTGATCGTTGGATT
QX1211_sago-2     ACTGAACTATGCTATTGGGAGTGAAGCGTTCAACAAGCCAAATAGATTGATCGTTGGATT
                  ************************************************************

QX1211_ppw-1      TGTTACTTCTCAACGAGTTGGTGGAAATCCAGATGTGAGTCATGTGTTACAGCTGCATAA
CB4856_ppw-1      TGTTACTTCTCAACGAGTTGGTGGAAATCCAGATGTGAGTCATGTGTTACAGCTGCATAA
N2_ppw-1          TGTTACTTCTCAACGAGTTGGTGGAAATCCAGATGTGAGTCATGTGTTACAGCTGCATAA
N2_sago-2         TGTTACTTCTCAACGAGTTGGTGGAAATCCAGATGTGAGTCATGTGTTACAGCTGCATAA
CB4856_sago-2     TGTTACTTCTCAACGAGTTGGTGGAAATCCAGATGTGAGTCATGTGTTACAGCTGCATAA
QX1211_sago-2     TGTTACTTCTCAACGAGTTGGTGGAAATCCAGATGTGAGTCATGTGTTACAGCTGCATAA
                  ************************************************************

QX1211_ppw-1      TCATATTTCATTTTTTCCAGTATCCAATATCAGTTGGATTTGCTGCAAATATGCTCAAGC
CB4856_ppw-1      TCATATTTCATTTTTTCCAGTATCCAATATCAGTTGGATTTGCTGCAAATATGCTCAAGC
N2_ppw-1          TCATATTTCATTTTTTCCAGTATCCAATATCAGTTGGATTTGCTGCAAATATGCTCAAGC
N2_sago-2         TCATATTTCATTTTTTCCAGTATCCAATATCAGTTGGATTTGCTGCAAATATGCTCAAGC
CB4856_sago-2     TCATATTTCATTTTTTCCAGTATCCAATATCAGTTGGATTTGCTGCAAATATGCTCAAGC
QX1211_sago-2     TCATATTTCATTTTTTCCAGTATCCAATATCAGTTGGATTTGCTGCAAATATGCTCAAGC
                  ************************************************************

QX1211_ppw-1      ATCATCAAAAGTTTGCTGGTGGATACGTGTATGTTCATCGCGATAGGGATGTTTTCGGAT
CB4856_ppw-1      ATCATCAAAAGTTTGCTGGTGGATACGTGTATGTTCATCGCGGTAGGGATGTTTTCGGAT
N2_ppw-1          ATCATCAAAAGTTTGCTGGTGGATACGTGTATGTTCATCGCGATAGGGATGTTTTCGGAT
N2_sago-2         ATCATCAAAAGTTTGCTGGTGGATACGTGTATGTTCATCGCGATAGGGATGTTTTCGGAT
CB4856_sago-2     ATCATCAAAAGTTTGCTGGTGGATACGTGTATGTTCATCGCGATAGGGATGTTTTCGGAT
QX1211_sago-2     ATCATCAAAAGTTTGCTGGTGGATACGTGTATGTTCATCGCGATAGGGATGTTTTCGGAT
                  ****************************************** *****************

QX1211_ppw-1      CCATCATCAAGGATACTCTCTTGACAATCTTCAAAACATGCACTGAGCAGCGCGGAAGAC
CB4856_ppw-1      CCATCATCAAGGATACTCTCTTGGCAATCTTCAAAACATGCACTGAGCAGCGCGGAAGAC
N2_ppw-1          CCATCATCAAGGATACTCTCTTGGCAATCTTCAAAACATGCACTGAGCAGCGCGGAAGAC
N2_sago-2         CCATCATCAAGGATACTCTCTTGACAATCTTCAAAACATGCACTGAGCAGCGCGGAAGAC
CB4856_sago-2     CCATCATCAAGGATACTCTCTTGACAATCTTCAAAACATGCACTGAGCAGCGCGGAAGAC
QX1211_sago-2     CCATCATCAAGGATACTCTCTTGACAATCTTCAAAACATGCACTGAGCAGCGCGGAAGAC
                  ********************** *************************************

QX1211_ppw-1      CAGATGATATCCTTTTGTATTTCAATGGAGTTTCCGAAGGTCAATTCTCAATGATCAACG
CB4856_ppw-1      CAGATGATATCCTTTTGTATTTCAATGGAGTTTCCGAAGGTCAATTCTCAATGATCAACG
N2_ppw-1          CAGATGATATCCTTTTGTATTTCAATGGAGTTTCCGAAGGTCAATTCTCAATGATCAACG
N2_sago-2         CAGATGATATCCTTTTGTATTTCAATGGAGTTTCCGAAGGTCAATTCTCAATGATCAACG
CB4856_sago-2     CAGATGATATCCTTTTGTATTTCAATGGAGTTTCCGAAGGTCAATTCTCAATGATCAACG
QX1211_sago-2     CAGATGATATCCTTTTGTATTTCAATGGAGTTTCCGAAGGTCAATTCTCAATGATCAACG
                  ************************************************************

QX1211_ppw-1      AGGAATTCAGTGCCCGAGTGAAGGAGGCGTGCATGGCATTCCAAAAGGAGGGAACCCCGC
CB4856_ppw-1      AGGAATTCAGTGCCCGAGTGAAGGAGGCGTGCATGGCATTCCAAAAGGAGGGAACCCCGC
N2_ppw-1          AGGAATTCAGTGCCCGAGTGAAGGAGGCGTGCATGGCATTCCAAAAGGAGGGAACCCCGC
N2_sago-2         AGGAATTCAGTGCCCGAGTGAAGGAGGCGTGCATGGCATTCCAAAAGGAGGGAACCCCGC
CB4856_sago-2     AGGAATTCAGTGCCCGAGTGAAGGAGGCGTGCATGGCATTCCAAAAGGAGGGAACCCCGC
QX1211_sago-2     AGGAATTCAGTGCCCGAGTGAAGGAGGCGTGCATGGCATTCCAAAAGGAGGGAACCCCGC
                  ************************************************************

QX1211_ppw-1      CATTCAGACCACACATCACCATCATCGCCTCATCAAAGGCTCACAACGAGCGTCTGTACA
CB4856_ppw-1      CATTCAGACCACACATCACCATCATCGCCTCATCAAAGGCTCACAACGAGCGTCTGTACG
N2_ppw-1          CATTCAGACCACACATCACCATCATCGCCTCATCAAAGGCTCACAACGAGCGTCTGTACA
N2_sago-2         CATTCAGACCACACATCACCATCATCGCCTCATCAAAGGCTCACAACGAGCGTCTGTACA
```

```
CB4856_sago-2      CATTCAGACCACACATCACCATCATCGCCTCATCAAAGGCTCACAACGAGCGTCTGTACA
QX1211_sago-2      CATTCAGACCACACATCACCATCATCGCCTCATCAAAGGCTCACAACGAGCGTCTGTACA
                   *********************************************************** 

QX1211_ppw-1       AATCCGACAAGGGACGTATTGTTAATTTGGAGCCGGGTACGGTCGTGGACCATACCATTG
CB4856_ppw-1       AATCCGACAAGGGACGTATTGTTAATTTGGAGCCGGGTACGGTCGTGGACCATACCATTG
N2_ppw-1           AATCCGACAAGGGACGTATTGTTAATTTGGAGCCGGGTACGGTCGTGGACCATACCATTG
N2_sago-2          AATCCGACAAGGGACGTATTGTTAATTTGGAGCCGGGTACGGTCGTGGACCATACCATTG
CB4856_sago-2      AATCCGACAAGGGACGTATTGTTAATTTGGAGCCGGGTACGGTCGTGGACCATACCATTG
QX1211_sago-2      AATCCGACAAGGGACGTATTGTTAATTTGGAGCCGGGTACGGTCGTGGACCATACCATTG
                   ************************************************************

QX1211_ppw-1       TGAGCAATGTCTACACTGAGTGGTATCATGCCTCAGCTGTTGCTCGCCAAGGAACTGCAA
CB4856_ppw-1       TGAGCAATGTCTACACTGAGTGGTATCATGCCTCAGCTGTTGCTCGCCAAGGAACTGCAA
N2_ppw-1           TGAGCAATGTCTACACTGAGTGGTATCATGCCTCAGCTGTTGCTCGCCAAGGAACTGCAA
N2_sago-2          TGAGCAATGTCTACACTGAGTGGTATCATGCCTCAGCTGTTGCTCGCCAAGGAACTGCAA
CB4856_sago-2      TGAGCAATGTCTACACTGAGTGGTATCATGCCTCAGCTGTTGCTCGCCAAGGAACTGCAA
QX1211_sago-2      TGAGCAATGTCTACACTGAGTGGTATCATGCCTCAGCTGTTGCTCGCCAAGGAACTGCAA
                   ************************************************************

QX1211_ppw-1       AGGCTACCAAGTTCACTCTCATCTTCACCACTAAAGCAGGCCCACAAGCTGAGCCATTGT
CB4856_ppw-1       AGGCTACCAAGTTCACTCTCATCTTCACCACTAAAGCAGGCCCACAAGCTGAGCCATTGT
N2_ppw-1           AGGCTACCAAGTTCACTCTCATCTTCACCACTAAAGCAGGCCCACAAGCTGAGCCATTGT
N2_sago-2          AGGCTACCAAGTTCACTCTCATCTTCACCACTAAAGCAGGCCCACAAGCTGAGCCATTGT
CB4856_sago-2      AGGCTACCAAGTTCACTCTCATCTTCACCACTAAAGCAGGCCCACAAGCTGAGCCATTGT
QX1211_sago-2      AGGCTACCAAGTTCACTCTCATCTTCACCACTAAAGCAGGCCCACAAGCTGAGCCATTGT
                   ************************************************************

QX1211_ppw-1       GGCATCTCGAGCAATTGACCAATGATCTTTGCTACGATCATCAGATTGTCTTCCATCCGG
CB4856_ppw-1       GGCATCTCGAGCAATTGACCAATGATCTTTGCTACGATCATCAGATTGTCTTCCATCCGG
N2_ppw-1           GGCATCTCGAGCAATTGACCAATGATCTTTGCTACGATCATCAGATTGTCTTCCATCCGG
N2_sago-2          GGCATCTCGAGCAATTGACCAATGATCTTTGCTACGATCATCAGATTGTCTTCCATCCGG
CB4856_sago-2      GGCATCTCGAGCAATTGACCAATGATCTTTGCTACGATCATCAGATTGTCTTCCATCCGG
QX1211_sago-2      GGCATCTCGAGCAATTGACCAATGATCTTTGCTACGATCATCAGATTGTCTTCCATCCGG
                   ************************************************************

QX1211_ppw-1       TCGGACTTCCCGTCCCACTGTACATTGCTGATCGTTACAGTCAACGTGGAGCAATGGTTC
CB4856_ppw-1       TCGGACTTCCCGTCCCACTGTACATTGCTGATCGTTACAGTCAACGTGGAGCAATGGTTC
N2_ppw-1           TCGGACTTCCCGTCCCACTGTACATTGCTGATCGTTACAGTCAACGTGGAGCAATGGTTC
N2_sago-2          TCGGACTTCCCGTCCCACTGTACATTGCTGATCGTTACAGTCAACGTGGAGCAATGGTTC
CB4856_sago-2      TCGGACTTCCCGTCCCACTGTACATTGCTGATCGTTACAGTCAACGTGGAGCAATGGTTC
QX1211_sago-2      TCGGACTTCCCGTCCCACTGTACATTGCTGATCGTTACAGTCAACGTGGAGCAATGGTTC
                   ************************************************************

QX1211_ppw-1       TTGCCGCCAATCAAGGGTAAGTGACAGTAAAATGTATACGACATCTCATTTATTATTTTC
CB4856_ppw-1       TTGCCGCCAATCAAGGGTAAGTGACAGTAAAATGTATACGACATCTCATTTATTATTTTC
N2_ppw-1           TTGCCGCCAATCAAGGGTAAGTGACAGTAAAATGTATACGACATCTCATTTATTATTTTC
N2_sago-2          TTGCCGCCAATCAAGGGTAAGTGACAGTAAAATGTATACGACATCTCATTTATTATTTTC
CB4856_sago-2      TTGCCGCCAATCAAGGGTAAGTGACAGTAAAATGTATACGACATCTCATTTATTATTTTC
QX1211_sago-2      TTGCCGCCAATCAAGGGTAAGTGACAGTAAAATGTATACGACATCTCATTTATTATTTTC
                   ************************************************************

QX1211_ppw-1       AGTCCAATCTACAATGAAGGACAAATCGATCTCGCGGCTACCAACAGTGCATATGGCTAC
CB4856_ppw-1       AGTCCAATCTACAATGAAGGACAAATCGATCTCGCGGCTACCAACAGTGCATATGGCTAC
N2_ppw-1           AGTCCAATCTACAATGAAGGACAAATCGATCTCGCGGCTACCAACAGTGCATATGGCTAC
N2_sago-2          AGTCCAATCTACAATGAAGGACAAATCGATCTCGCGGCTACCAACAGTGCATATGGCTAC
CB4856_sago-2      AGTCCAATCTACAATGAAGGACAAATCGATCTCGCGGCTACCAACAGTGCATATGGCTAC
QX1211_sago-2      AGTCCAATCTACAATGAAGGACAAATCGATCTCGCGGCTACCAACAGTGCATATGGCTAC
                   ************************************************************

QX1211_ppw-1       GGAGAGAAGAAGCTCTTCACCACTCGTTTCAATGCATAATTATCTGTACTCCTCCGTTTC
```

```
CB4856_ppw-1       GGAGAGAAGAAGCTCTTCACCACTCGTTTCAATGCATAATTATCTGTACTCCTCCGTTTC
N2_ppw-1           GGAGAGAAGAAGCTCTTCACCACTCGTTTCAATGCATAATTATCTGTACTCCTCCGTTTC
N2_sago-2          GGAGAGAAGAAGCTCTTCACCACTCGTTTCAATGCATAATTATCTGTACTCCTCCGTTTC
CB4856_sago-2      GGAGAGAAGAAGCTCTTCACCACTCGTTTCAATGCATAATTATCTGTACTCCTCCGTTTC
QX1211_sago-2      GGAGAGAAGAAGCTCTTCACCACTCGTTTCAATGCATAATTATCTGTACTCCTCCGTTTC
                   ************************************************************

QX1211_ppw-1       TTTTACTTGGCTGTATAATGTTTTATTTCCGTAAACCCAGAATACTTTCGCTATGATTGT
CB4856_ppw-1       TTTTACTTGGCTGTATAATGTTTTATTTCCGTAAACCCAGAATACTTTCGCTATGA-TGT
N2_ppw-1           TTTTACTTGGCTGTATAATGTTTTATTTCCGTAAACCCAGAATACTTTCGCTATGATTGT
N2_sago-2          TTTTACTTGGCTGTATAATGTTTTATTTCCGTCAACCCAGAATACTTTCGCTATGATTGT
CB4856_sago-2      TTTTACTTGGCTGTATAATGTTTTATTTCCGTAAACCCAGAATACTTTCGCTATGATTGT
QX1211_sago-2      TTTTACTTGGCTGTATAATGTTTTATTTCCGTAAACCCAGAATACTTTCGCTATGATTGT
                   ******************************** ********************** ***

QX1211_ppw-1       ATATTTTTTAGCATTTGGTCATTGTAATTTGTAATTATTATCGGAATGGATAAAATGATT
CB4856_ppw-1       ATATTTTTTAGCATTTGGTCATTGTAATTTGTAATTATTGTCGGAATGGATAAAATGATT
N2_ppw-1           ATATTTTTTAGCATTTGGTCATTGTAATTTGTAATTATTGTCGGAATGGATAAAATGATT
N2_sago-2          ATATTTTTTAGCATTTGGTCATTGTAATTTGTAATTATTGTCGGAATGGATAAAATGATT
CB4856_sago-2      ATATTTTTTAGCATTTGGTCATTGTAATTTGTAATTATTGTCGGAATGGATAAAATGATT
QX1211_sago-2      ATATTTTTTAGCATTTGGTCATTGTAATTTGTAATTATTGTCGGAATGGATAAAATGATT
                   *************************************** ********************

QX1211_ppw-1       TAAGAATAAAATGCATCGCTTCTTGGACAA--------
CB4856_ppw-1       TAAGAATAAAATGCATCGCTTCTTGGACAA--------
N2_ppw-1           TAAGAATAAAATGCATCGCTTCTTGGACAA--------
N2_sago-2          TAAGAATCAAATGCATCGCTTCTTGGACAATTCCTGTT
CB4856_sago-2      TAAGAATTAAATGCATCGCTTCTTGGACAATTCCTGTT
QX1211_sago-2      TAAGAATAAAATGCATCGCTTCTTGGACAATTCCTGT-
                   ******* **********************
```
