# Supplementary figures and images for: Diversification of small RNA pathways underlies germline RNA interference incompetence in wild Caenorhabditis elegans strains
Source: Genetics. 2023 Oct 22;226(1):iyad191. doi: 10.1093/genetics/iyad191 (PMC10763538; doi:10.1093/genetics/iyad191)

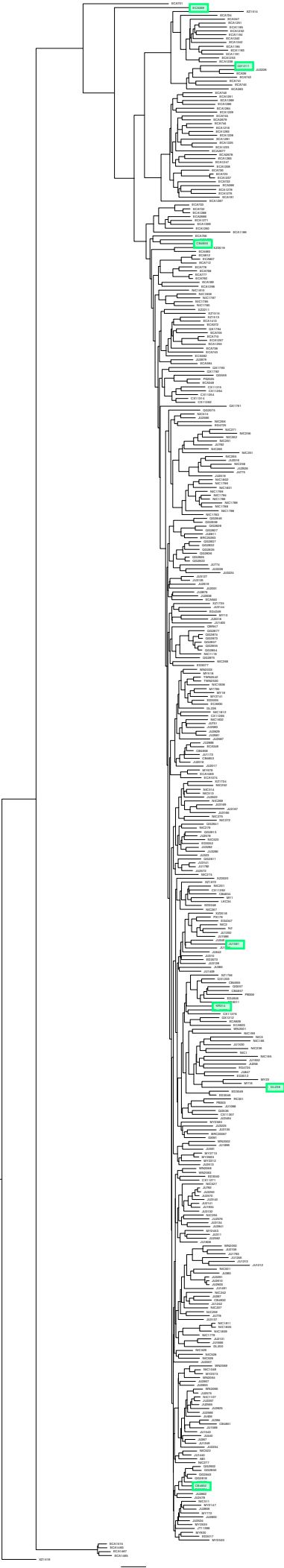

Supplement: iyad191_Supplementary_Data [file iyad191_supplementary_data.zip › Figure_S9_GENETICS-2023-306148.pdf]
